# Country-report pattern corrections of new cases allow accurate 2-week predictions of COVID-19 evolution with the Gompertz model

**DOI:** 10.1038/s41598-024-61233-w

**Published:** 2024-05-11

**Authors:** I. Villanueva, D. Conesa, M. Català, C. López Cano, A. Perramon-Malavez, D. Molinuevo, V. L. de Rioja, D. López, S. Alonso, P. J. Cardona, C. Montañola-Sales, C. Prats, E. Alvarez-Lacalle

**Affiliations:** 1 Department of Physics, Universitat Politècnica de Catalunya (BarcelonaTech), 08860 Castelldefels, Spain; 2 Department of Information and Communication Technologies, Universitat Pompeu Fabra, 08018 Barcelona, Spain; 3 Nuffield Department of Orthopaedics, Rheumatology and Musculoskeletal Sciences (NDORMS), University of Oxford, Oxford, UK; 4 Medical Image Processing Lab, École Polytechnique Fédérale de Laussane, Geneva, Switzerland; 5 Microbiology Department, Laboratori Clínic Metropolitana Nord, Hospital Universitari Germans Trias i Pujol, Institut Universitari Germans Trias i Pujol (IGTP), Badalona, Catalonia Spain; 6 Departament of Genetics and Microbiology, Universitat Autònoma de Barcelona, Cerdanyola, Catalonia Spain; 7 Biomedical Research Networking Centre in Respiratory Diseases CIBERES, Instituto de Salud Carlos III, Madrid, Spain; 8 Department of Quantitative Methods, IQS School of Management, Universitat Ramon Llull, 08017 Barcelona, Spain; 9 Comparative Medicine and Bioimage Centre of Catalonia (CMCiB), Fundació Institut d’Investigació en Ciències de la Salut Germans Trias i Pujol, 08916 Badalona, Spain

**Keywords:** Epidemiology, Computational models

## Abstract

Accurate short-term predictions of COVID-19 cases with empirical models allow Health Officials to prepare for hospital contingencies in a two–three week window given the delay between case reporting and the admission of patients in a hospital. We investigate the ability of Gompertz-type empiric models to provide accurate prediction up to two and three weeks to give a large window of preparation in case of a surge in virus transmission. We investigate the stability of the prediction and its accuracy using bi-weekly predictions during the last trimester of 2020 and 2021. Using data from 2020, we show that understanding and correcting for the daily reporting structure of cases in the different countries is key to accomplish accurate predictions. Furthermore, we found that filtering out predictions that are highly unstable to changes in the parameters of the model, which are roughly 20%, reduces strongly the number of predictions that are way-off. The method is then tested for robustness with data from 2021. We found that, for this data, only 1–2% of the one-week predictions were off by more than 50%. This increased to 3% for two-week predictions, and only for three-week predictions it reached 10%.

## Introduction

The appearance of SARS-CoV-2 in 2019 in the Wuhan region of China^1–3^ has presented an enormous challenge for hospitals and Intensive Care Units (ICUs) around the world^4,5^. A significant number of asymptomatic and pre-symptomatic cases have helped to propagate the COVID-19 disease^6,7^ with high hospitalizations and assisted support requirements^4,8^, unless large vaccination coverage is achieved. This situation has specifically affected elder people and susceptible population^9,10^. Significant increases in COVID-19 cases lead to a high rate of hospitalization and ICU’s use, which require long-term support^11^. This condition has led to the collapse of the standard hospital function in regions where the incidence reached high values (typically 2% fourteen-day incidence values or higher) until the arrival of the omicron variant and vaccination campaigns^12,13^. Therefore, it is important to develop predictive tools that can forewarn increases in demand for services due to COVID-19. In these circumstances, hospitals need to mobilize resources from both within and, if needed, outside the hospital. Personal time shifts, the opening of new areas for COVID-19 treatment, reduction of non-urgent activities, among others, need to be planned, preferably 2 weeks in advance.

The development of these predictive tools requires two fundamental analyses. First, regions establish a pattern of hospitalization charge and discharge from the number of detected cases, vaccination coverage, and the local characteristics of the therapeutic effort. Health authorities have very accurate data on the ratio of detected cases that need hospitalization and, eventually, ICUs^14^, except for the very first stages of a new variant with higher transmission. They also have exact and local values for the structural delay between the development of COVID-19 symptoms and hospitalization needs^15^. Given the typical uncertainty between a reported case and hospitalization, around 2–10 days^16^, health authorities need the number of symptomatic cases today to predict the hospital situation in the following days. Unfortunately, PCR tests require time to be requested, performed and introduced in the information system (IS)^15^. Even in the case of fast antigen tests, there are delays between symptoms and physician visits. These delays in consolidating data prevent proper hospital utilization prediction unless the number of issues can be known accurately some days in advance.

The need for highly accurate one-week prediction tools for COVID-19 cases from consolidated reported cases has triggered the development of highly calibrated short-term prediction models. They can be divided into mechanistic, artificial intelligence (AI), and empirical growth models.

In the first category, mechanistic models (SEIR-type models) are typically compartment models that divide a population into Susceptible, Exposed, Infectious and Recovered. The transitions between these compartments are governed by differential equations describing how individuals move from one state to another over time. The model parameters include the transmission rate, the incubation period, and the recovery rate, among others. These models provide insights into the potential course of an outbreak, the impact of interventions (such as vaccination or social distancing), and the overall dynamics of the disease within a population. In addition, they are employed for direct short-term prediction^17^ with the incorporation of quarantined individuals^18^ or to evaluate the role of social distancing^19^ or different local legislative and social environments^20–22^.

In the second category, AI-driven time series analysis of disease cases allows for short-term predictions on the number of cases^23,24^ or derived hospitalizations because they do not depend on the susceptibility of the individuals. The Autoregressive Integrated Moving Average (ARIMA) or Long Short-Term Memory (LSTM) algorithms are broadly used in that matter. Usually, machine learning strategies can be employed to improve the short-term predictions developed with time series analysis^25,26^, offering a robust framework for forecasting disease cases or hospitalizations and refining public health decision-making processes.

Finally, growth models are typically used to describe growth processes of different types, particularly epidemics^27^. They have been particularly applied to the short-term prediction of COVID-19 cases or hospitalizations because their dynamics do not depend on the susceptibility of the individuals and can account for any non-pharmacological intervention. Examples of growth models are the logistic model^28^ or the model employed in this report, the Gompertz model^29^. The Gompertz model, successfully fitted to data from different countries, provides reasonably accurate forecasts 5–10 days ahead^30,31^. Different versions of these models, including the one presented here, have been employed during the pandemic to perform short-term predictions from the evolution of the number of daily cases. For example, 28 and 29 independent models have been employed to forecast the evolution of cases, respectively, in the different states of the United States of America^32^ and in the different countries in Europe^33^. A key feature of all these models is that the ground truth data they must fit entail only daily diagnoses. In this way, there is a more general split between models that aim to predict multiple countries from only case count cases from models that can integrate different inputs in a given country or region. For example, the mobility data can explain much of the growth rate^34^. Furthermore, climate^35^, social interactions^36^ wastewater^37^, or a combination of them together with hospitalization, prevalence, or past deaths have been employed to enhance the performance of the predictions^38^. One of the main problems previous approaches face is the unreliable weekly dynamics of daily reported cases. They present notable differences between labor days and weekends in most countries^39–41^. It is commonly associated with different activities in primary care, where most cases are detected nowadays^42^, and laboratory or Information System delays due to the weekend break. We will show, however, that daily patterns are more extensive and complicated. We will show that unveiling them provides valuable information useful for producing accurate data that can not be directly obtained with weekly averages. The basic idea is that, once the patterns are known, the last data of a given weekday provides information for the future that is diluted when performing weekly averages.

In the present paper, we perform predictions using case counts after analyzing European patterns of reported cases and correcting them. We focus on the daily patterns observed during the European waves at the end of 2020 and 2021. We will use 2020 as the testing ground of the method to make it self-contained, and then we update the reporting pattern for 2021 to test the method’s robustness. We show that direct empirical data on the reporting patterns of each country increases the short-term accuracy of the predictions. We also show that prediction must be tested for local robustness. Predictions that change significantly when the model's internal parameters are changed should be considered unstable. They have worse prediction accuracy. Allowing for a 30% error in the number of new cases, we show a success rate for the last trimester of 2021 higher than 90% with one-week predictions and close to 80% for two-week predictions once unstable predictions are filtered out. More importantly, one or two-week predictions that are off by more than 50% are rare (less than 3%). Finally, we compare the model’s prediction for different European countries against the performance of other models participating in the European Hub of forecasters^33^ over one and two-week horizons.

## Methods

### Data source and pre-processing

We obtain the historical data series of new daily cases for European countries with more than 1 million inhabitants from the WHO database^43^. We analyze two batches of data. First, we use European data from the 1st of September to the end of November 2020, encompassing the second and third waves, depending on the country. We fully analyse our prediction methods with these data from 2020. Then, we use the same dates for 2021 to check that the best prediction method analyzed and developed for 2020 is also reliable in 2021, in a different stage of the European pandemic associated with different dominant variants.

To assess the reliability of the data series, we checked the number of days where cases reported were zero for each country. We have found that the daily cases in Denmark, Norway, Sweden, and Cyprus are unreliable due to significant gaps in the data. These gaps include extended periods of null entries on various dates rather than occasional interruptions. As a result, we have concluded that the most appropriate course of action is to disregard this data. A full description is provided in Supplementary Table S1.

We group the other countries as those providing systematic data (see Supplementary Table S1) and those having a one or two-day gap, generally related to a given holiday. Countries with only one gap in data present cases reported the next day of the missing data. It is relatively straightforward that the cases accumulated due to holidays, so the series can be easily corrected by distributing those extra cases to the previous day. Nevertheless, they still fail to provide accurate data for some days, and it is challenging to redistribute cases properly. Therefore, we do not include predictions for holidays.

Countries present a characteristic pattern of weekly oscillations. We must stress here that the data series of each country does not correspond to the day the cases were detected, but the day the cases were reported. In other words, if fewer cases are detected during the weekend but countries report them with a one or two-day delay, the data series will tend to present fewer cases on Monday or Tuesday and not during the weekends.

Finally, as discussed in the introduction, Gompertz-type predictions are macroscopic models^30^ that can not consider the effect of small fluctuations as individual-based modelling or spatially extended models of SEIR can address^44^. Whenever the cases are very low in a country, the evolution can be determined by the local characteristics of the particular outbreak. This is outside of the model’s scope. Therefore, we take a minimum weekly average of 100 daily cases. In the first batch, we find two countries, Estonia and Latvia, in which most days analyzed do not exceed our daily case limit, so they are excluded from the study of this batch.

### Gompertz-like prediction models

The time series of new cases has been used to forecast the epidemics in the short term using a global Gompertz fit.

1 $$G\left( t \right) = G_{o} + K {\text{exp}}\left[ { - \ln \left( {\frac{K}{{N_{b} }}} \right)e^{{ - \alpha \left( {t - t_{o} } \right)}} } \right]$$

where K corresponds to the final number of cases, *N*_*b*_ is the initial number of cases for time *t*_*o*_, and parameter *a* is the rate of decrease in the initial exponential growth^30^. Note that *K*, *N*_*b*_, and *a* are related through the initial exponential growth of the number of cases^30^. The idea behind the prediction is to use the Gompertz function to obtain parameters *K*, *G*_*o*_, *N*_*b*_, *t*_*o*_, and *a* that minimize the global fit of the time series. A different *G*_*j,tf*_ (*t*) is associated for each country *j* using data for the fit from *t *_*f −N*_ until date *t *_*f*_ . This function is then used to obtain the prediction for *t* > *t*_*f*_.

The study considers only the daily cases which allows for obtaining the accumulated number of cases. Due to this limitation, we fit first the accumulated cases and then the daily cases. The accumulated number of cases is more robust regarding fluctuations than the daily cases. However, the daily cases may provide more information. Although there are different options regarding which penalty function to use to fit the parameters of the Gompertz function, here, we analyze two simple penalization methods, studying how they impact the accuracy of the predictions. Notice that both methods are used to validate that predictions are improved when the daily pattern is considered.

In the first method (A), we minimize the deviation of the Gompertz-fit in terms of cumulative cases (*CC*). This is, we minimize the following error:

2 $$e_{A} = \mathop \sum \limits_{{d \in N_{pred} }} \frac{{\left| {CC\left( d \right) - CC_{pred} \left( d \right)} \right|}}{CC\left( d \right)}$$

where *N*_*pred*_ is the number of days for which a prediction is obtained and *CC*_*pred*_ are the predicted cumulative cases.

In the second method (B), we minimize the deviation in the number of new cases (*C*) in addition to the deviation in accumulated cases. The error function to minimize reads:

3 $$e_{B} = e_{A} + \mathop \sum \limits_{{d \in N_{pred} }} \frac{{\left| {C\left( d \right) - C_{pred} \left( d \right)} \right|}}{C\left( d \right)}$$

**Figure 1:**
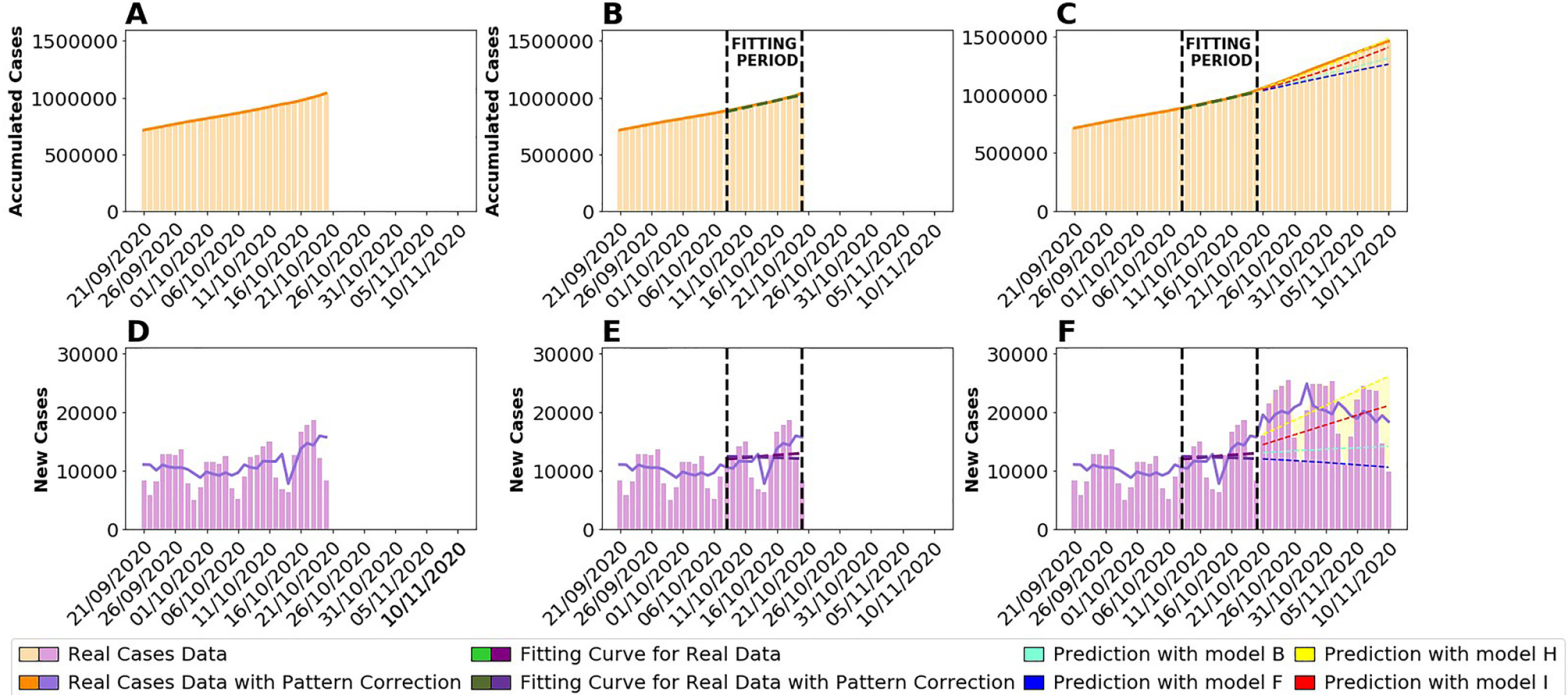
Pipeline of the process using the example of the Spanish prediction of cases on October 20th 2020. The top panels show the case data in a cumulative format, while the bottom row shows the data in a new cases format. (**A**) and (**D**) Portrait the real evolution of COVID-19 cases, in bars, and the same data with pattern correction applied, in a line. (**B**) and (**E**) Show the data that is represented in the first column, as well as the fitting period, delimited by vertical black dashed lines, and the actual fitting curves, both for real data and corrected data, in colored dashed lines. Panels (**C**) and (**E**) show the prediction curves for each model, in colored dashed lines. In panel (**E**), the yellow shadow shows the difference between the actual real data and the prediction data for model Hallmark (H) model H. The new cases prediction curve is obtained transforming the accumulated fitting curve to new cases by subtracting the data from date *t* to the data from date *t*−1. Legend indicates the specific color meaning and association with our four different models.

We then have two minimization methods and we can activate and deactivate the daily pattern correction—all in all, we consider four different possibilities in the manuscript. We name them: model B (Baseline) for the one using the raw data without correction and the cumulative minimization; model I (Introduction of Patterns) when we also use the minimization of the cumulative function but introduce the weighted time series where the daily patterns of the data reports are introduced; model F (Fallback) when we use the minimization of the new cases but without introducing the daily pattern correction to the case data; and finally, model H (our Hallmark) where we use minimization of the new cases reported with the cases count data corrected using the daily reporting pattern in each country. A graphical description of the complete pipeline, including a representation of the curve-fitting, the minimization, and the prediction evaluation processes, is shown in Fig. 1.

It is important to note that the number of days *N* we include in our predictions is critical. The value of *N* must be long enough to detect the tendency of epidemics, but not so long that it includes the effects of previous waves. In the manuscript, we use first the value of two weeks (*N* = 14) because it seemed like a good compromise between these two constraints. However, testing that the model predictions do not change significantly if we take *N* to be slightly more or less than two weeks is essential. In other words, predictions must not change much if we take between one week and a half weeks and close to three weeks of previous data. We address this issue in the “Results” section, showing that taking *N* = 14 provides the best overall prediction.

### Pattern analysis

To unveil clearly the weekly pattern of detection and reporting, we compute the seven-day moving average of the series at a given day *t*: $n_{{7}} (t) = \sum^{t + {3}}_{i=t-3} n(i)/{7}$, where *n*(*i*) is the data point of new cases on day *i* following the same idea presented in. We assess the ratio between new cases in a certain day, *n*(*t*), and the corresponding 7-day moving average value, *n*_7_(*t*), as this day’s ratio $w(t) = n(t)/n_{{7}} (t)$. If we group these ratios by each day of the week, we can identify if a daily pattern exists and evaluate it. The closer to 1, the less deviation from the average. The further from 1, the higher the deviation from the average.

**Figure 2:**
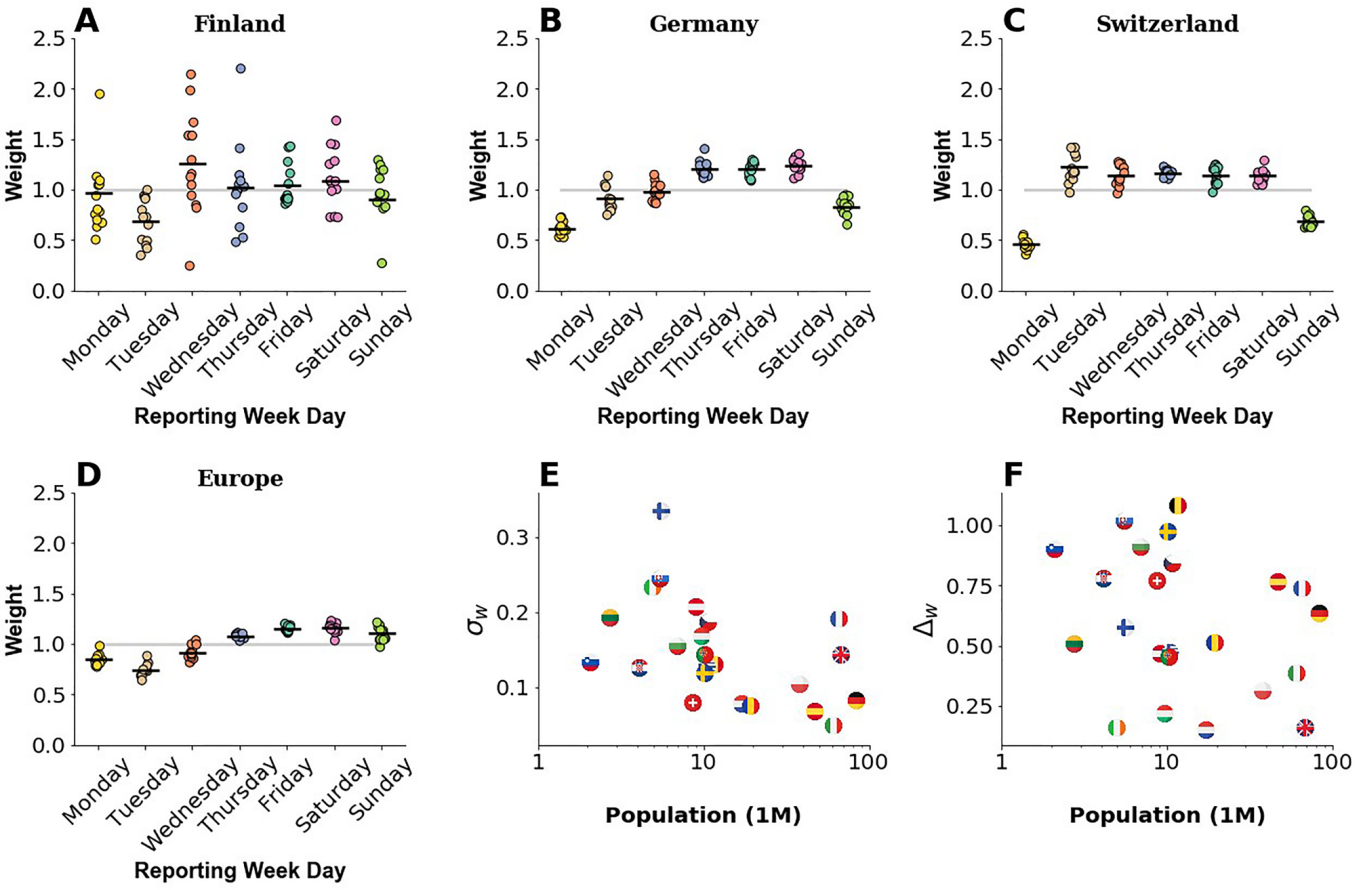
Weekly reporting pattern for Finland, Germany, Switzerland, and Europe computed between September 1, 2020, and November 30, 2020. The weekly standard deviation of the pattern *σ*_*w*_ is presented in panel (**E**). Panel (**F**) shows the difference between the maximum and minimum average daily weight ∆*w* for each country, identified with its flag.

Figure 2 presents examples of these ratios for each day of the week in three representative countries plus the aggregated data for all UE + EFTA + UK countries for the 2020 data. The horizontal line indicates the average value of the ratio, while discrete points show the different ratios obtained depending on the week being analyzed. We can observe that the weekend effect in the European aggregate data is reflected in a drop in Mondays and Tuesdays reported cases. We observe roughly 20% under-reporting each day. Switzerland and Germany present different lags in reporting, so the affected days are different. In Switzerland, reported cases to WHO drop on Sundays and Mondays. The figure also shows the significant differences in the dispersion of the data. In Finland, there are substantial fluctuations in the ratio from week to week. Figure 2 also shows the ratio's weekly average standard deviation σw as a measure of this dispersion. As expected, we observe a clear correlation between less population and more fluctuations. Finally, we scatter plot the difference between the maximum and minimum average daily weight ∆*w* as a function of the population. We can observe that this difference does not depend on the population but on the reporting idiosyncrasies of each country. The daily pattern of all countries under study is in Supplementary Fig. S1.

### Corrected series of new cases

The unveiling of clear daily patterns allows us to give a global weight *W* to each weekday *d* (such as Monday) depending on the country *j*:

4 $$W_{j,d} = \frac{1}{{N_{w} }}\mathop \sum \limits_{t \in T_d} w_{j}\left( t \right)$$

where *N*_*W*_ is the number of weeks in the series, *T*_*d*_  is the set of days in the series that correspond to a certain weekday *d*, and *w*_*j*_(*t*) the day’s ratio for a certain country *j*.

From these global weights, we can construct a corrected series of new cases for each country. If we call *n *_*j*_(*t*) the number of new cases in a country *j* for a given day *t*, we construct the series cases *C*_*j*_ as:

5 $$C\left( j \right) = \frac{{n_{j} \left( t \right) }}{{W_{j,d} }}$$

where *d* is the day of the week associated with day *t*. So we now have the original series of new cases *n *_*j*_(*t*) and the corrected series of new cases *C*_*j*_(*t*), which is different in each country *j* according to their particular reporting pattern.

### Methodological summary

We give here a more step-by-step general description of the methods used. We first consider the daily case counts in European countries from the WHO database that do not present important gaps in the data. We divide the daily reported cases by the corresponding 7-day moving average and then compute the mean of these ratios for each weekday to derive a global weight for every day of the week. Using these global weights, we construct a corrected series of new cases for each country, dividing the number of new cases by their corresponding weights. In this way, we correct the weekly reporting pattern of each country, as it has been recently done to monitor the flu^45^. Subsequently, we fit each country’s reported data to a Gompertz function to generate the prediction of new cases. Finally, we evaluate the performance of the 4 possible models that can be created by combining the following options: First, using the non-corrected series or the weekly pattern-corrected series as the series of daily cases. Second, minimizing the deviation of the Gompertz-fit in terms of cumulative cases (computed as the cumulative sum of daily cases) or both cumulative and daily cases.

## Results

### Performance analysis of the prediction models

We produce three-week predictions of new cases for European countries from 1st September until 28th of November 2020 on a bi-weekly basis (Saturdays and Tuesdays). We do these predictions using a first method to minimize the cumulative error before the prediction date to compute the best Gompertz fit, and a second method where we add the minimization of the error of new cases before the prediction date (see “Methods”). We also perform each one using the bare list of cases obtained from the database and the corrected list of cases where daily report patterns are detected and corrected (see “Methods”). Therefore, we use a total of four different models to perform the predictions.

**Figure 3:**
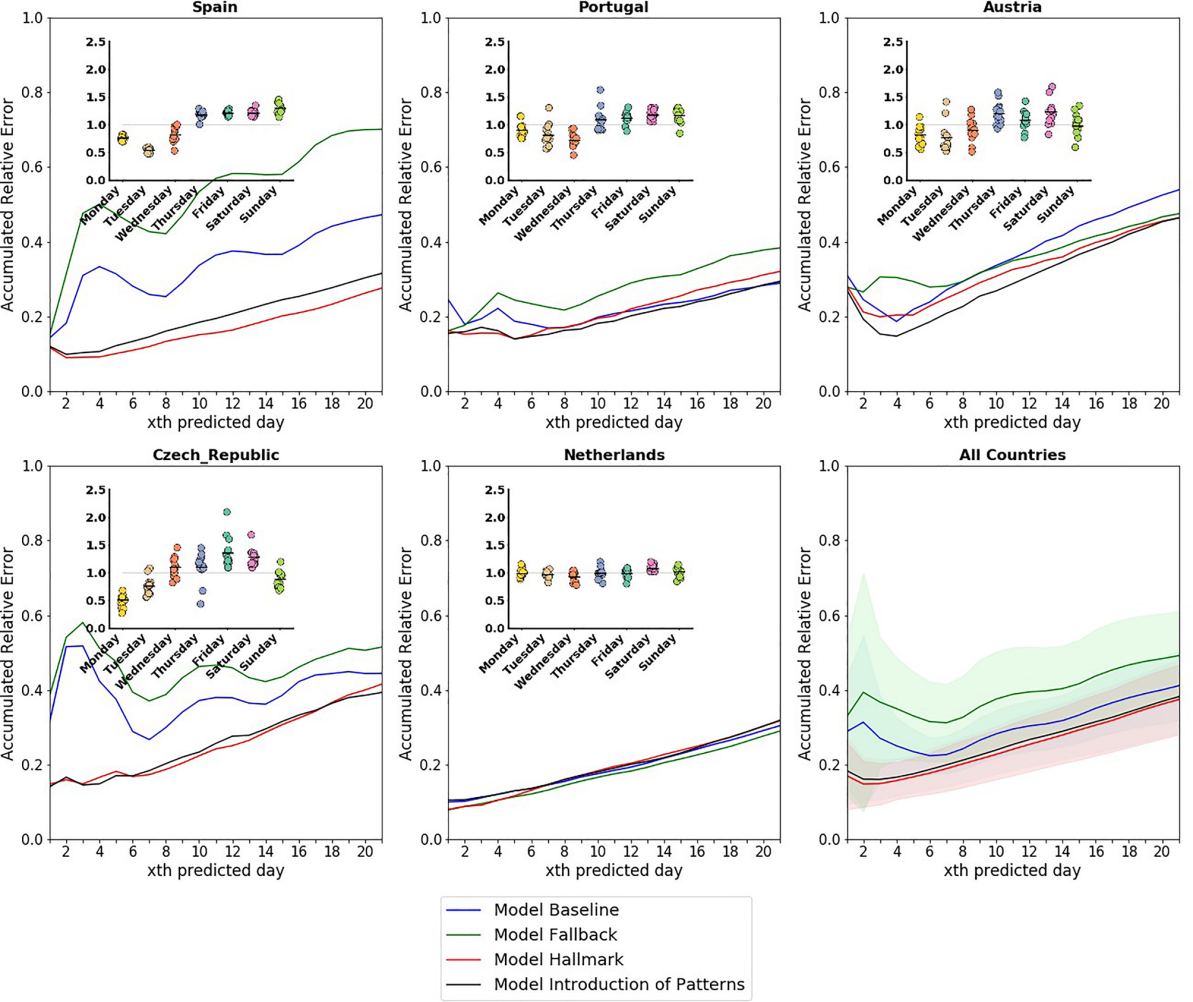
Accumulated relative error of each model for Spain (**A**), Portugal (**B**), Austria (**C**), Czech Republic (**D**), and the Netherlands (**E**), presented along with the reporting pattern of each of the countries. Panel (**F**) shows the averaged accumulated relative error for all considered European countries together with its standard deviation, shown in shades. Model B considers the minimization for accumulated cases and no daily pattern correction, model F minimizes new cases and no daily pattern correction, model H minimizes accumulated cases and daily pattern correction, and model I minimizes new cases and daily pattern correction. The accumulated relative error is calculated by accumulating the relative error each day; that is, the accumulated relative error for the xth day is the sum of relative errors from day 0 up to day x−1.

Figure 3 shows the average deviation (MSE) of our prediction of new cases from reality as a function of the number of days from the prediction date for different key representative countries and for the prediction including all countries (see the last panel). Globally, the best performance, as observed in the MSE averaged across the list of European countries, is for our Hallmark model. The second best also uses the correction of daily patterns but a different minimization method. We observe how introducing the daily pattern correction improves the predictions. Typical one-week errors are around 20%, increasing to 25–40% when predictions are two to three weeks ahead.

Correcting the daily pattern of cases described in the methodology significantly improves the predictions, especially within a one-week horizon. Without them, errors typically have a minimum after one week, once the effect of the pattern is less significant. However, correcting the implicit bias generated by the reporting clarifies the tendency of infections. The daily pattern correction does eliminate not only this minimum but also improves the prediction across the board. Correcting this bias improves predictions in most but not all countries.

The selected countries in Fig. 3 show some exciting characteristics depending on the particular country. Countries like Spain and the Czech Republic improve dramatically when the daily pattern corrects the case data series. Predictions are highly inaccurate when the bare case count is used, no matter the minimization procedure. On the other hand, for some countries like the Netherlands, all models produce similarly good predictions. A small subset of countries have slightly better results with a different model than our Hallmark model. The few countries for which the best model is clearly not the Hallmark (model H) are Austria, Belgium, Finland, Ireland, Lithuania, Slovakia, and Slovenia (see Supplementary Fig. S2 to check all countries). Typically, the second best model overall, model I (Introduction of patterns), is the best model in these countries. There is only one country where the introduction of the daily patterns worsens the prediction: Finland.

**Figure 4:**
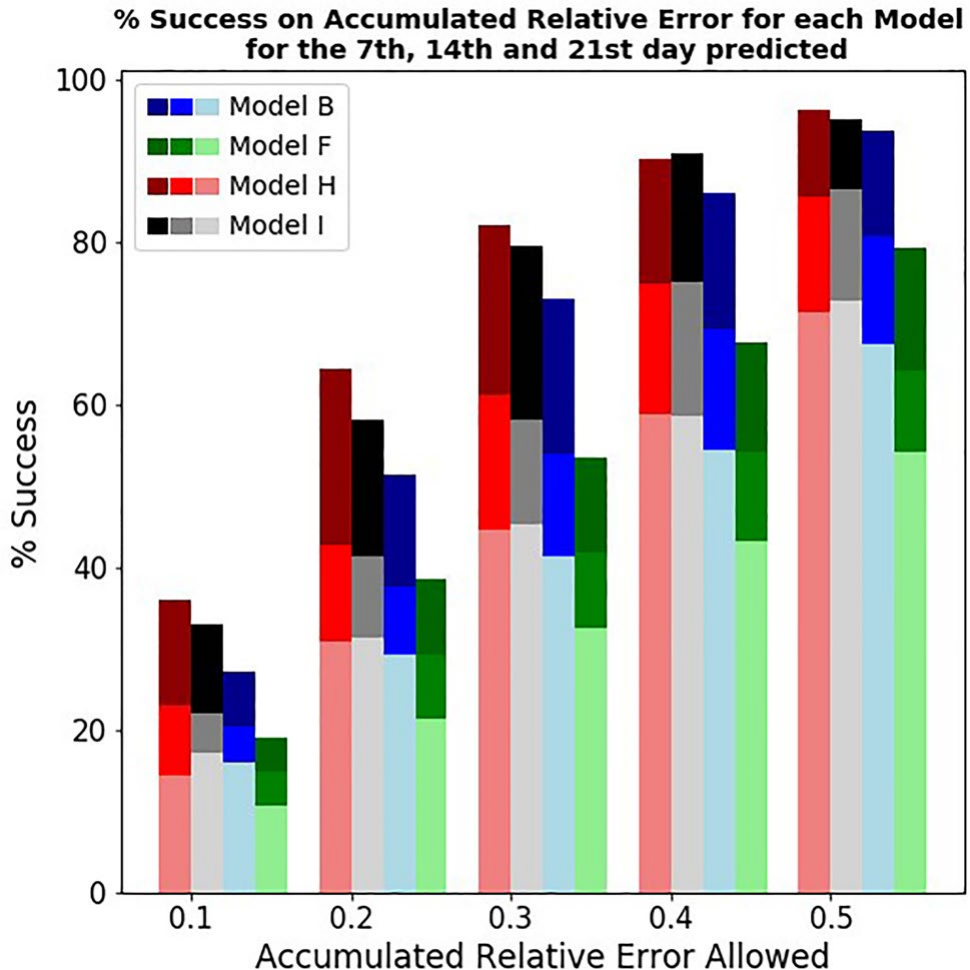
The success rate for each model when allowing a certain error (see x-axis) for the averaged accumulated relative error among all the considered European countries (23). This metric has been computed by only considering the accumulated relative error of the 7th, 14th, and 21st predictions. For each model, the lightest shade shows the results for prediction at three weeks, while the darkest shade shows the results for one-week prediction.

In any case, the daily pattern correction is particularly relevant for one-week predictions. We further analyze the accuracy of predictions by checking the presence of outliers. We want to analyze how many predictions were accurate using 10% intervals. Figure 4 shows for the four models how many of the predictions were accurate within 10%, 20%, 30%, 40%, and 50% to the actual number of new cases reported during 7 days, 14 days, and 21 days after the prediction date. Notice that predictions at 7 days are always more accurate than at 14 and 21. Model F is the worst across the board. The Hallmark (H) model markedly improves the number of predictions with errors below 20%.

Figure 4 shows the number of predictions that were off by a large margin. That means it visualizes how many times the prediction was off by more than 50%. For example, model H had roughly a 95–96% success rate in making predictions with errors below 50%. This result means slightly more than 4–5% of our one-week predictions using model H had a huge mistake. With 4–5% of predictions markedly off, we can call these predictions outliers since they represent a minority of our predictions. However, Fig. 4 shows that the number of predictions that fail substantially increases when we go to a 2-week or 3-week prediction. For 2-week predictions, predictions failing to have below 50% error are close to 15%, while for 3-week they increase markedly to 30%.

We notice that both models H and I have a similar number of outliers. They make a similar number of very wrong predictions. Model F is particularly lacking, with more than 40% predictions off. In this sense, large mistakes of the predictions at the three-week horizon using model F are really not outliers but a common feature.

In order to make reasonable predictions useful for the healthcare system, is more important to avoid outliers than to increase the accuracy of the most precise predictions. In other words, the penalty for making one big mistake in the prediction is high. In this sense, model H is also the best one in having high accuracy in cases. It also has lower number of predictions with large errors. Given that we have detected this relevance of the daily pattern in the correction of outliers, we proceed to analyze if we can detect the reasons behind the outliers that remain present in using method H. We aim to understand the robustness of model H. In other words, we want to check if this small set of 4–5% erroneous predictions could have been captured and some pre-screening filter applied.

### Bias and robustness analysis of model Hallmark. Reduction of the prediction outliers

We first check if our predictions are biased in the sense that a previously accelerating or decelerating growth in the epidemics would make our predictions over or underestimate the tangible outcome. To test if this is the case, we use the effective growth rate as

6 $$\rho \left( t \right) = \frac{{C_{j} \left( {t + 1} \right) + C_{j} \left( {t + 2} \right) + C_{j} \left( {t + 3} \right)}}{{C_{j} \left( {t - 2} \right) + C_{j} \left( {t - 3} \right) + C_{j} \left( {t - 4} \right)}}$$

following the same procedure to describe the epidemics empirically as found in^46^ and we can compute if the epidemics are accelerating or decelerating using the change of this growth rate:

7 $${\Delta }\rho \left( t \right) = \rho \left( {t + 4} \right) - \rho \left( {t - 6} \right)$$

**Figure 5:**
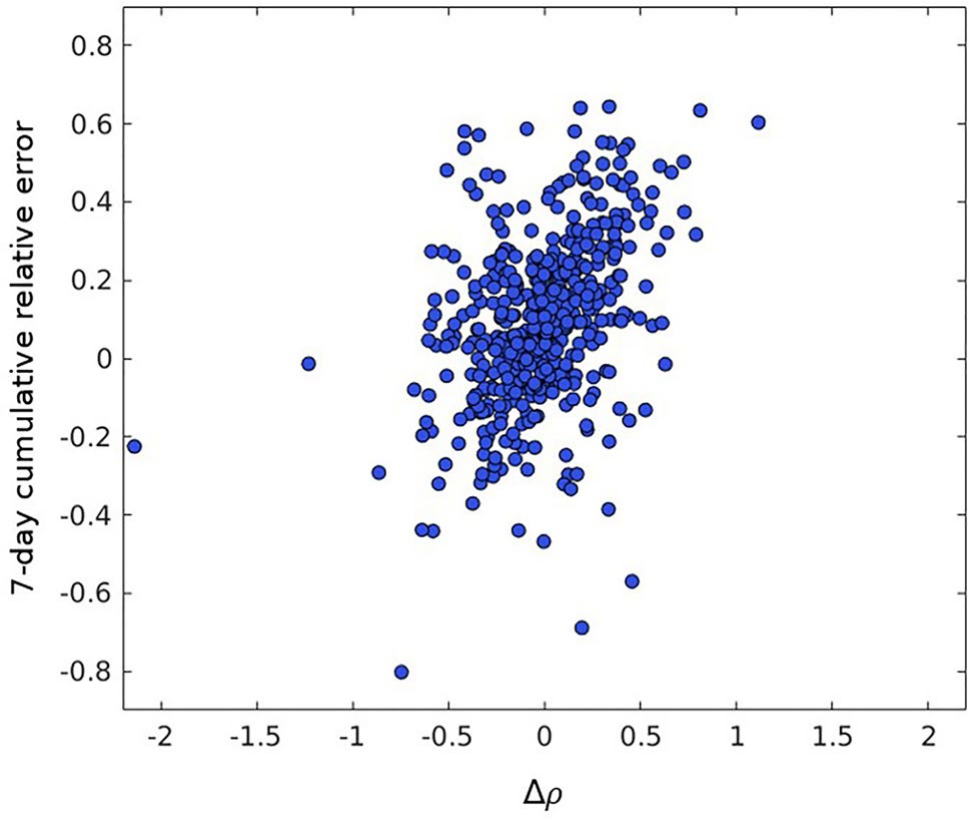
Difference between the primary reproduction number (*ρ*) obtained 4 days after prediction and 6 days before as a function of the 7-day cumulative relative error for all countries and dates considered.

In each of our predictions, we compute its ∆*ρ* to check for any correlation between the acceleration of the epidemics and the sign of our error to test for possible bias. Figure 5 shows the 7-day relative cumulative error for all our predictions as a function of ∆*ρ*. The correlation coefficient is 0.16, indicating our model does not present an important bias. We cannot improve our prediction by minimizing this type of bias in our prediction model.

**Figure 6:**
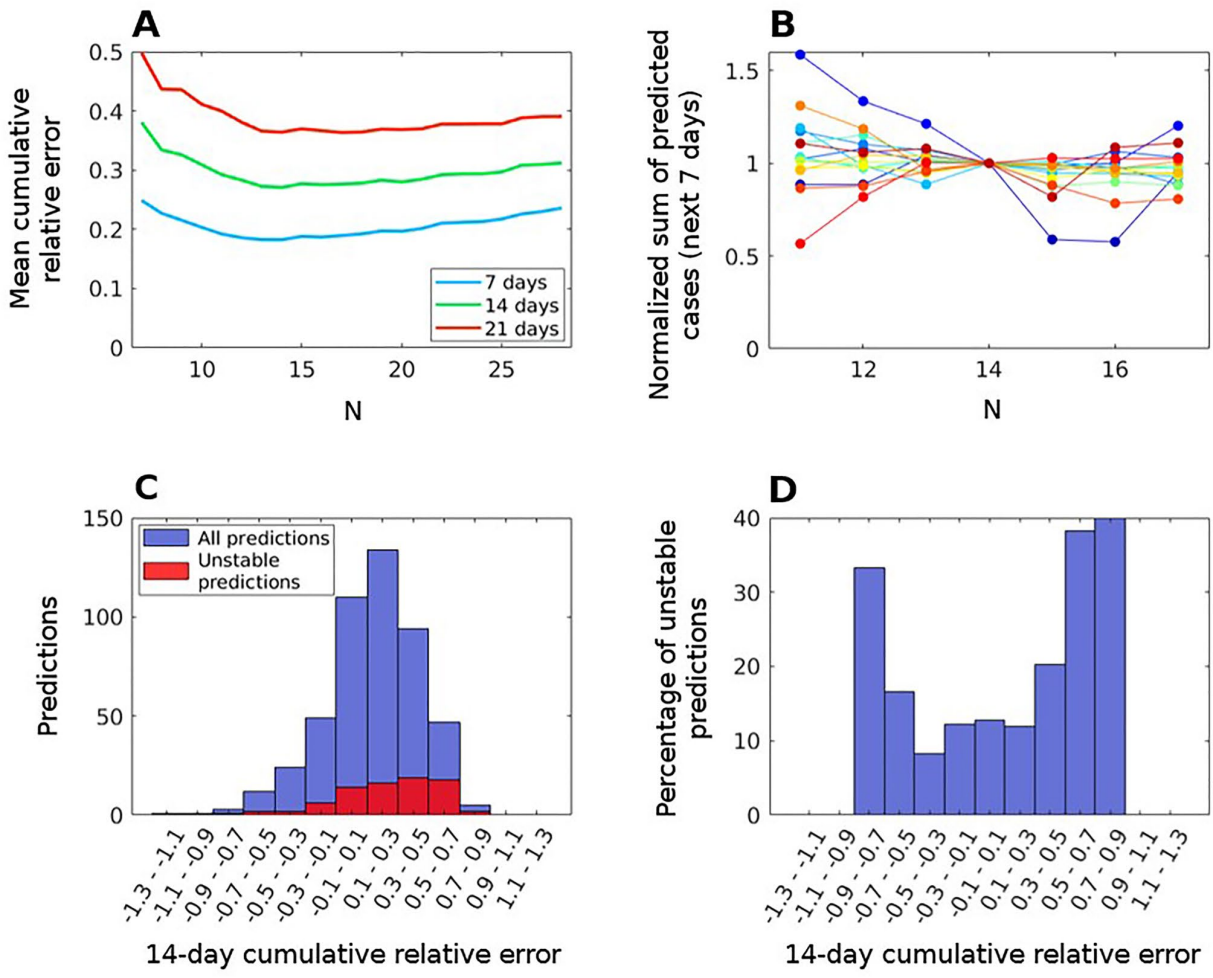
Evaluation of the instability of the model. (**A**) Variation of the mean cumulative relative error for the next 7 (blue), 14 (green) and 21 (red) days predicted cases for all countries and dates analyzed. (**B**) Normalized sum of the cases predicted for the next 7 days in Slovenia, obtained using a different number of days to fit the model. Different colors correspond to different predictions. (**C**) 14-day cumulative relative error distribution for all countries and dates predicted (blue) and only unstable predictions (red). (**D**) Percentage of unstable predictions present in each 14-day cumulative error band for all countries and dates predicted.

However, we find that a small set of our predictions is highly susceptible to changes in the number of past days used to make the prediction. We find a subset of our predictions that changes enormously depending on the parameter *N*. We show in Fig. 6 that the lowest errors in our predictions are obtained using a two-week window in the past. However, some of these predictions change abruptly when *N* is changed by just one day. In panel B of Fig. 6, we show as an example all our predictions for Slovenia with those for *N* = 14 normalized at one to observe the relative change in our prediction as we modify *N*. As we can see, most of our predictions are robust within 10%, but one of them shows an abrupt change, by more than 40%, just by changing from *N* = 14 to *N* = 15. It is thus clear that choosing a past history of *N* = 14 is a very good option for the model’s prediction, as long as the prediction outcome is not extremely sensitive to selecting precisely this value.

We now proceed to analyze if those highly unstable predictions have a worse prediction profile than the average. We consider those predictions that change by more than 25% when a day is included or removed in our data (i.e., *N* increases or decreases by one day) to be highly unreliable. Similarly, some predictions clearly tend to increase and decrease strongly as *N* increases. They are not stable either. We take any prediction that changes more than 35% upon changes in the value of *N* from 12 to 18 as having a clear non-stable tendency. Normally, this tendency continues up to *N* = 21 making the outcome too sensitive to the criteria used to incorporate information from the past. We select both of them as unreliable predictions and analyze if they provide worse accuracy than stable predictions.

Panels (C) and (D) of Fig. 6 show that this is indeed the case. In panel (C), the distribution of all errors is indicated together with how many are unstable in each bracket, while panel (D) shows the percentage of the predictions in each error bracket belonging to the unstable predictions. From our sample of 480 predictions, unstable predictions represent 20% of the total percentage, but they overpopulate our worst predictions being 30–40% of those predictions. They are indeed more unreliable across the board.

**Figure 7:**
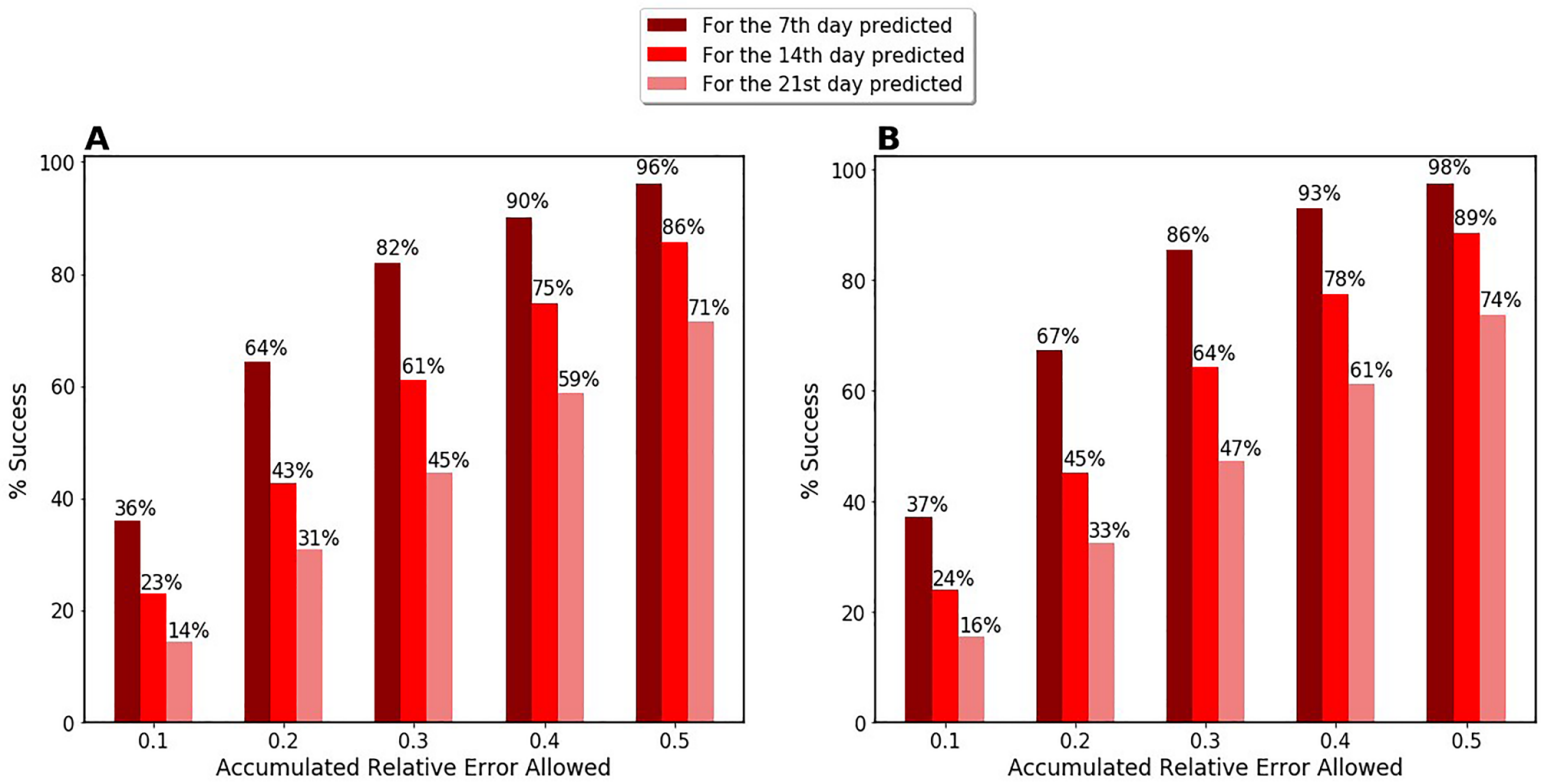
The success rate for the 7th, 14th, and 21st predictions given allowed an accumulated relative error for model H (Hallmark) for 2020 data. Panel (**A**) considers all of our predictions, whether they are stable or unstable, while in panel (**B**) unstable predictions are disregarded.

We proceed to check if removing this 20% of our prediction has a powerful effect on our accuracy to check if the penalty of missing some of our predictions is worth it. Figure 7 shows the differences between the success rates both considering and disregarding those unstable predictions. As expected, there is no significant improvement in the accuracy of the predictions done with less than a 30% error, the real change in percentage terms, comes in the outliers for one and two-week predictions. The success rate for a one-week prediction goes from 96 to 98%. With this filter, only 2% of the total predictions are outliers. This gives very huge confidence level to our prediction model in the one-week forecast framework. This fact is a rather impressive result because half of the outliers in one-week predictions are eliminated. Similarly, the improvement is remarkable in the two-week prediction where the elimination of the unstable predictions eliminates one third of the total outliers. The success rate moves from below 86% to 89%. For a three-week prediction, the improvement is only marginal.

### Reliability. Predictions in 2021

**Figure 8:**
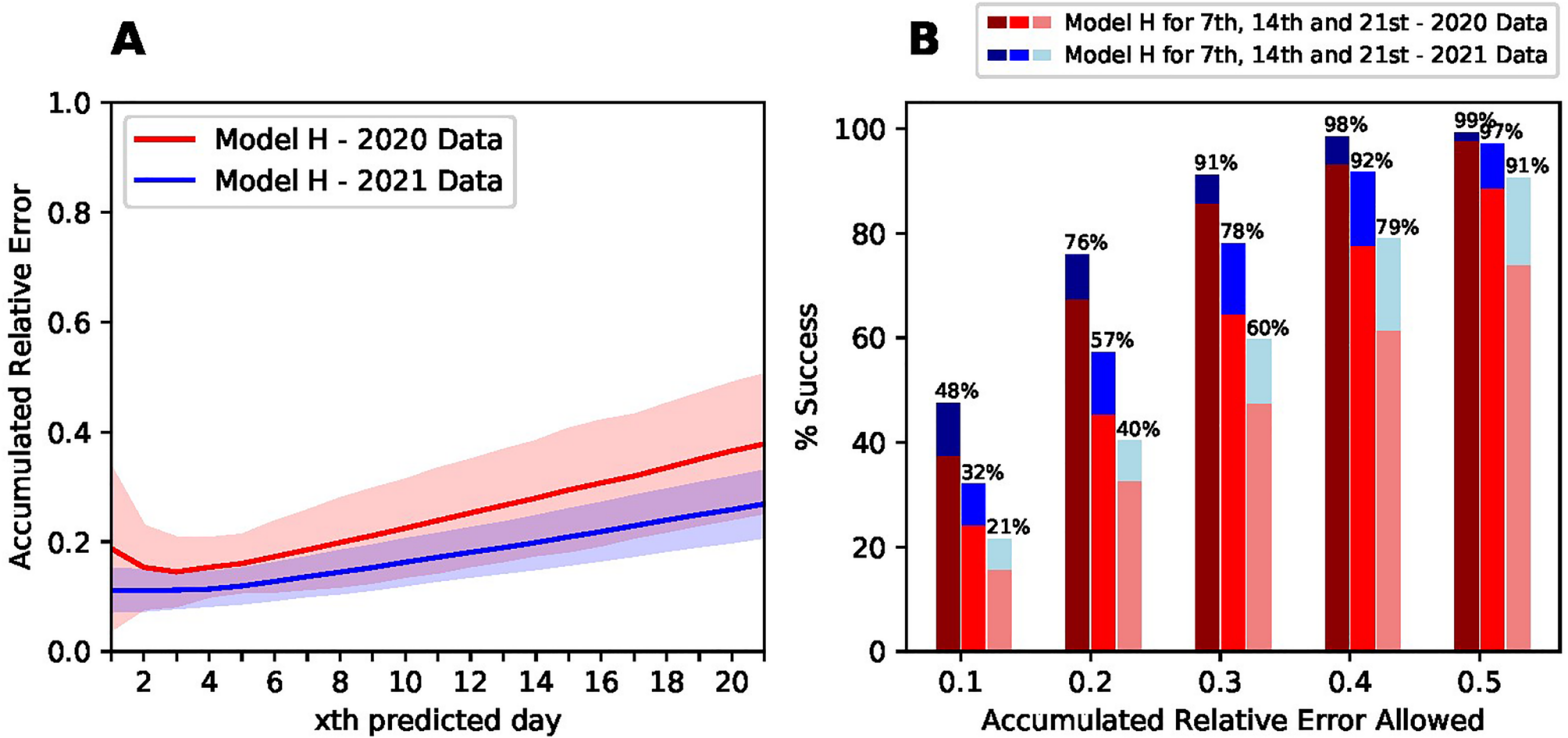
Left: Average relative error accumulated up to the number of days indicated in the x-axis from all European data in 2020 and in 2021. Right: Success rate as the number of predictions that have an error below the indicated in the x-axis. For each allowed relative error, each bar represents predictions at 1, 2, and 3 weeks, respectively. The different colors indicate the prediction for 2020 (reds) and for 2021 (blue). Success rate is higher for 2021 than for 2020 in each one of the prediction horizons and in each one of the relative errors allowed. The numbers indicated are the success rates for 2021 (blue bars).

We checked that the process we have developed with data from 2020 works properly with data from 2021. Figure 8 compares the best model H with data from 2020 already presented and the same dates for the end of 2021. The average relative error in the biweekly predictions across all countries is systematically lower for the different predictions horizon from 1 day to 3 weeks. This reduction in errors is pretty consistent across all countries (see Supplementary Fig. S3) for all prediction horizons.

We proceed to analyze if this reduction in the average errors also reduces the number of prediction outliers, this is, the number of predictions that are off by more than 50%. We analyze again one, two and three-weeks predictions. The right panel of Fig. 8 shows how the ratio of successful predictions below a given error is systematically larger in 2021 predictions. Especially relevant is the increase in the low level of 2-week predictions that were off by less than 50% going up from 11% (89% success rate) to only 3% (97% success rate). The Supplement Material shows that a lot of countries do not present any predictions far-off. Some countries, like Germany, Romania, or Portugal have highly accurate two-week predictions with typical errors around 10%. It is also very remarkable the large increase in the success rate in three-week predictions in 2021. While in 2020, only 60% of the three-week predictions had an error below 40%, the same number for 2021, jumped to close to 80% rendering 4 in 5 predictions reasonable accurate three weeks in advance.

### Comparative performance of the model

We compare the average error of our model in the context of the hub of European pedictions^33^, to which we contributed by submitting weekly predictions elaborated with the model described above. These predictions were done with daily cases and deaths separately, being each one an independent prediction. Here, we only describe the case count prediction. Given that the data came from different countries with different health care systems and protocols, the case count, in each country, represented a different unknown fraction of the real propagation of the disease. However, we could correct for the weekly underreported days, following the procedure described here so that we have a good signal preprocessing. We also checked the robustness of each prediction and did not submit a prediction in case it was unstable, following the protocol explained in the previous sections.

**Figure 9:**
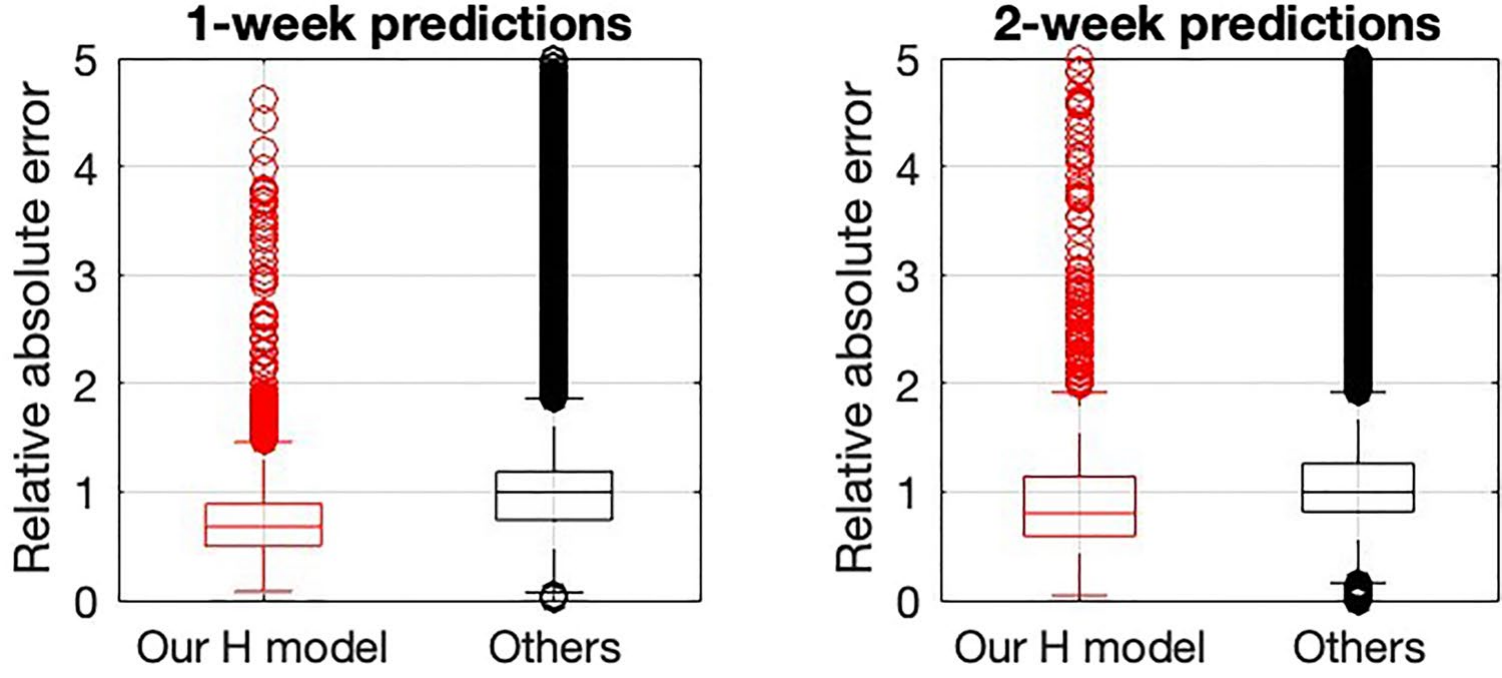
Left: Average relative absolute error in the subsets of predictions done for the European Hub of models (See Sherratt et al.^33^) for our 1-week horizon in comparison with all the other 28 models from the 6th of September 2021 to the 26th of December 2022. We use our H model using the protocol to disregard predictions that are not robust. Right: same as left but for a two-week prediction horizon.

In Fig. 9, we show the distributions of absolute errors for all 1-week and 2-week predictions, comparing the ones obtained with our model with the predictions individually submitted by other contributing models. We observe how our model is indeed a good one, showing that our method had a lower error than the average of other models. However, all models were relevant and important since, globally, the ensemble prediction built from the median of all models beat any of the individual models. Although a particular model can behave better than the ensemble in a particular epidemiological context, evaluating the ensemble’s global performance provides more robust prediction results. As described in^33^, this is probably because different models capture different features given the various approaches. Our model focused on not giving outliers and being reliable, while others focused more on accuracy in the different confidence intervals. In any case, our approach performed well compared with other models with the exact prediction purpose and using the same ground truth data.

## Discussion

Two-week prediction of case numbers in European countries can be performed with a Gompertz model with a high accuracy level and a low number of important mistakes for different waves associated with different variants as long as a given set of criteria for the prediction is established. First, the number of cases must lead to general community transmission, otherwise, unpredictable chance plays a very important role. Second, accurate pattern recognition of the reporting structure of the country must be taken into account otherwise important errors are introduced in the prediction. And finally, the model can present instabilities in the prediction. Sometimes, the pattern of cases is highly ambiguous regarding its structure. This is detected by analyzing how the prediction changes when the number of data from the past is slightly changed.

Gompertz and Logistic models have been employed to describe and predict the cumulative cases of COVID-19^47,48^, which have been compared with other predictive models like the Logistic and Artificial Neural Networks models^49^. Here, we have found that roughly 20% of our bi-weekly predictions present this kind of instability in the analysis of the second European COVID-19 in late 2020. These predictions are an important fraction of the overall large mistakes. Filtering these predictions out allows us to reduce the number of one-week predictions that are off by more than 50% to just 1 out of 50 predictions. More importantly, the two-week horizon prediction is only off in 1 out of 10 predictions.

We also observe that the accuracy of the predictions was higher in 2021 than in 2020. The rate of success in the three-week prediction was significantly higher in 2021 than in 2020, rendering this medium-term prediction rather accurate. In 2021, nearly 80% of the three-week predictions had a relative error below 40% and more than half an error below 30%. The improvement is across the board in most countries. Given that the prediction method relies on an accurate assessment of the tendencies in the case count, the higher accuracy is necessarily related to a better evolutionary fit to a Gompertz-like evolution with fewer sudden changes. We should notice that the dominant variant in Europe was different at the end of 2020 than in 2021, and the level of non-pharmacological measures was different. This might lead to different persistence in the dynamics. Analyzing which one was more relevant is out of the scope of this manuscript, but it is worth pointing out that, as the evolution of the dynamics depends less on changes in non-pharmacological interventions, the short and medium-term dynamics can become more predictable.

We have estimated the mean absolute error, equivalent to the mean absolute percentage error (MAPE) but from 0 to 1, and quantified the error rate below a certain threshold. Such results can be compared with the MAPE error obtained from other techniques for evaluating other types of quantifications in COVID-19 predictions. For example, techniques commonly used are based on the approach of time series, like the Auto Regressive Integrated Moving Average (ARIMA) or the Nonlinear Autoregression Neural Network (NARNN)^50^ or, based on deep learning models such as multi-head attention Long-short term memory (LSTM), or convolutional neural network (CNN) with the Bayesian optimization algorithm^51^. Such methods have been employed during the pandemic to estimate the number of cumulative cases with similar error levels in the predictions.

This remarkable fact allows for very good predictions for health officials since the model does not take into account any other information than the past structure of cases. This makes the prediction very robust to smooth changes in the behavior of the epidemics. It also allows the model to be applied to a wide range of different countries, as long as there is a sufficiently long history of data cases, showing a good performance when compared with other models used in the European Hub of models^33^ developed to predict reported cases during the epidemic. In this sense, our model performed actually better in 2021 than in 2020 despite important differences in the level of non-pharmacological interventions. Changes in mobility or social interactions due to seasonality in behavioral patterns, or seasonality in terms of weather are not incorporated in the model, but, as long as changes are not abrupt, the model captures its effects in the structure of cases. We have shown that the best way to make predictions is to use roughly two-weeks of past data, and that the model must be robust when incorporating three weeks of data from the past. So, as long as interaction or new variant introduction changes have a time scale of a couple of weeks, our model should capture it and make reasonable predictions.

Our approach makes an important tool for health officials when deciding future healthcare needs in hospitalization. Since the severity of the disease manifests roughly one week after diagnosis, predicting cases with reasonable accuracy two weeks in advance, allows a three-week window for preparation. There is an important limitation. New variants can render past relationships between cases and hospitalizations obsolete, as the comparisons between Omicron and Delta severity show^52^. This predictive approach has been continuously used in the group for predictions at the European level and for Catalan Health authorities. However, its effects on hospitalization do need a constant update since the severity of the disease changes as the number of susceptible drops.

Furthermore, another limitation of our model should be specified. Whenever the protocols for detecting cases change our data is not homogeneous and the prediction necessarily fails. Whenever a drastic change of criteria for counting cases is introduced, as has happened in the past once the level of susceptible population decreased a lot after the Omicron wave, our model must be discontinued for some weeks. Once the data is again systematic and there are some weeks with common criteria across time for detection, it can be used again.

### Supplementary Information

Supplementary Information.

## Data Availability

Case count data is open and provided by WHO. These data and all the codes for prediction elaboration and filtering are in https://github.com/InmaV/COVID-19-predictions.
