# Supplementary material for: Country-report pattern corrections of new cases allow accurate 2-week predictions of COVID-19 evolution with the Gompertz model
Source: Sci Rep. 2024 May 11;14:10775. doi: 10.1038/s41598-024-61233-w (PMC11087483; doi:10.1038/s41598-024-61233-w)
Supplement: Supplementary file 1 — Supplementary Information. [file 41598_2024_61233_MOESM1_ESM.pdf]

Country-report pattern corrections of new cases allow accurate two-week predictions of COVID-19 evolution with the Gompertz model Supplemental Material

I. Villanueva[1,2,+], D. Conesa[1,+], M. Català[3], C. López Cano[1], A. Perramon-Malavez[1], D. Molinuevo[4], V. López de Rioja[1], D. López[1], S. Alonso[1], P. J. Cardona[5,6,7], C. Montañola-Sales[8], C. Prats[1,9], and E. Alvarez-Lacalle[1,*]

[1,*]Department of Physics, Universitat Politècnica de Catalunya (BarcelonaTech), Castelldefels, 08860, Spain
[2]Department of Information and Communication Technologies, Universitat Pompeu Fabra, Barcelona, 08018, Spain
[3]Nuffield Department of Orthopaedics, Rheumatology and Musculoskeletal Sciences (NDORMS), University of Oxford, Oxford, UK.
[4]Medical Image Processing Lab.École Polytechnique Fédérale de Laussane, Genève, Switzerland
[5]Microbiology Department, Laboratori Clnic Metropolitana Nord, Hospital Universitari Germans Trias i Pujol, Institut Universitari Germans Trias i Pujol (IGTP), Badalona, Catalonia, Spain
[6]Departament of Genetics and Microbiology, Universitat Autnoma de Barcelona, Cerdanyola, Catalonia, Spain
[7]Biomedical Research Networking Centre in Respiratory Diseases CIBERES, Instituto de Salud Carlos III, Madrid, Spain
[8]IQS School of Management. Department of Quantitative Methods. Universitat Ramon Llull, Barcelona, 08017, Spain
[9]Comparative Medicine and Bioimage Centre of Catalonia (CMCiB), Fundaci Institut dInvestigaci en Cincies de la Salut Germans Trias i Pujol, Badalona, 08916, Spain
[*]enric.alvarez@upc.edu
[+]these authors contributed equally to this work

Introduction

In this supplemental material we show, first, how countries have been filtered to know whether their reporting data is reliable to make the study or not (Supplementary Table S1). Then, the reporting pattern (Supplementary Figure S1) and performance analysis of the four different prediction methods (Supplementary Figures S2 to S4) can be observed for every country which passed the initial filter. Finally, Supplementary Table S2 represents which prediction days have been considered for every country and what predictions are considered unstable to compare the analysis when considering all the predictions or rejecting the unstable ones.

1 Country filtering: no reporting data

A list of countries belonging to EU, EFTA and UK is shown in Supplementary Table S1 with their population and number of days with no report of new cases. Those countries with more than 2 days without reporting cases are coloured in red, meaning these countries are discarded for our study due to the unreliability of the data: Cyprus, Denmark and Norway. The countries presenting one or two days without reporting data are coloured in yellow, but the data series of these countries is easily corrected by distributing the extra cases of the next day. Finally, the two countries coloured in orange (Estonia and Latvia) present such low amounts of cases that most days do not reach the new cases threshold explained in the methods sections of the main manuscript to take them into account.

Country	Population	Std. deviation	Peak-to-peak diff	No report days
Austria	9,006,400	0.2067	0.4631	0
Belgium	11,589,616	0.1298	1.0774	0
Bulgaria	6,948,445	0.2122	0.9081	2
Croatia	4,105,268	0.1252	0.7782	0
Cyprus	1,207,361	0.4927	0.3253	5
Czech Republic	10,708,982	0.1864	0.8416	0
Denmark	5,792,203	0.3466	0.7869	6
Estonia	1,326,539	0.2981	0.5967	1
Finland	5,540,718	0.3655	0.5737	1
France	65,273,512	0.1899	0.7372	0
Germany	83,783,945	0.0819	0.6318	0
Greece	10,423,056	0.1262	0.4682	0
Hungary	9,660,350	0.1669	0.2180	0
Ireland	4,937,796	0.2324	0.1562	0
Italy	60,461,828	0.0490	0.3823	0
Latvia	1,886,202	0.3287	0.8454	1
Lithuania	2,722,291	0.1913	0.5068	0
Netherlands	17,134,873	0.0744	0.1460	0
Poland	37,846,605	0.1500	0.3093	1
Portugal	10,196,707	0.1863	0.4504	1
Romania	19,237,682	0.1255	0.5121	1
Slovakia	5,459,643	0.2441	1.0163	0
Slovenia	2,078,932	0.1327	0.8995	0
Spain	47,026,000	0.0670	0.7630	0
Sweden	10,099,270	0.1175	0.9697	0
Norway	5,421,242	0.1692	2.3742	26
Switzerland	8,654,618	0.0784	0.7682	0
United Kingdom	67,886,004	0.1425	0.1582	0
Europe	527,862,990	0.0493	0.4129	0

Supplementary Table S1. List of countries with their population, standard deviation and peak-to-peak difference of data of new cases of COVID-19 and number of days without reporting new cases. The eliminated countries for the study are coloured in red and orange due to unreliability of data, while countries with yellow background are marked as countries with days without case reports but with such a little amount that their series are easily corrected.

2 Pattern analysis

The ratio between new cases on the day t and the 7-day moving average value, $n_7(t)$ is defined as

$$w(t) = \frac{n(t)}{n_7(t)} \tag{1}$$

In Supplementary Figure S1 we show the reporting patterns, depending on the day of the week, of the 23 countries taken into account in this study.

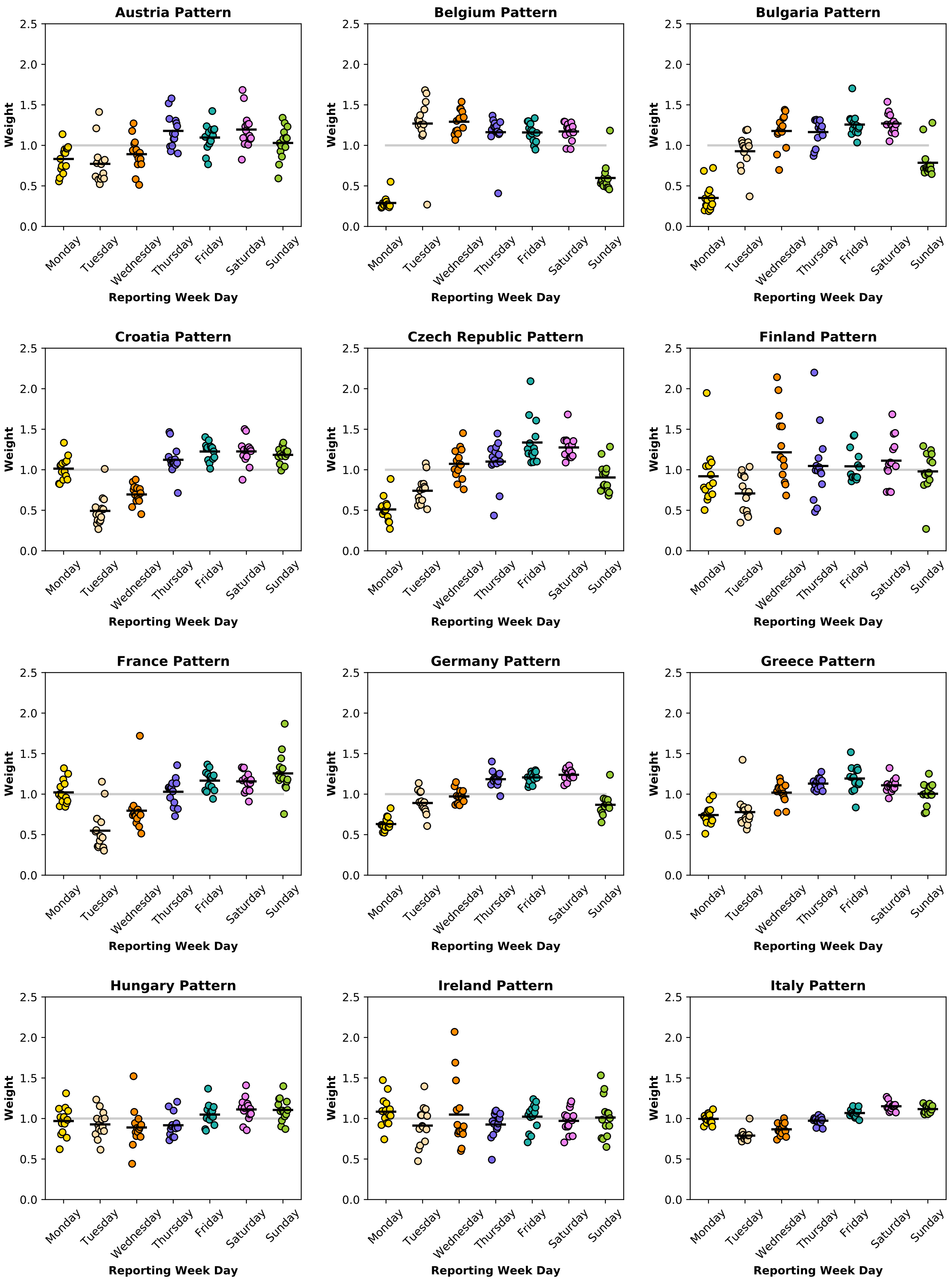
Austria Pattern
Belgium Pattern
Bulgaria Pattern
Croatia Pattern
Czech Republic Pattern
Finland Pattern
France Pattern
Germany Pattern
Greece Pattern
Hungary Pattern
Ireland Pattern
Italy Pattern
Weight
0.0
0.5
1.0
1.5
2.0
2.5
Monday
Tuesday
Wednesday
Thursday
Friday
Saturday
Sunday
Reporting Week Day

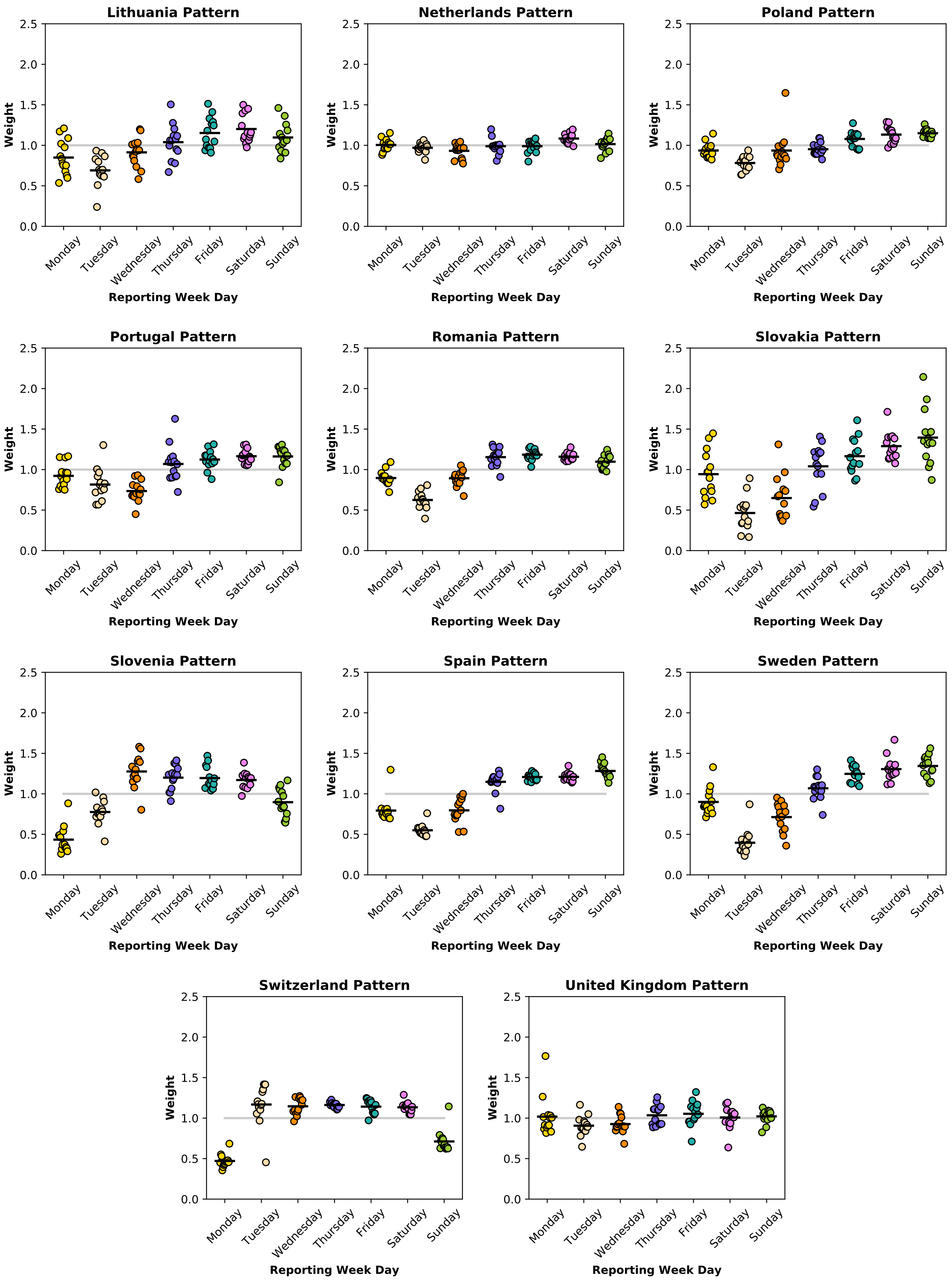

Supplementary Figure S1. Reporting patterns of the 23 EU+EFTA+UK countries that remain after applying the first filter in Supplementary Table S1.

3 Performance analysis of the prediction methods

Four different models of prediction have been used in this study to fit the Gompertz function when considering different minimization functions:

- Model B (Baseline): minimizes the error in accumulated cases.
- Model F (Fallback): minimizes the error in new cases.
- Model H (Hallmark): minimizes the error in new cases with corrected data due to their daily patterns.
- Model I (Introduction of Patterns): minimizes the error in accumulated cases with corrected data due to their daily patterns.

The accumulated relative error of the prediction fit while using the four different models can be observed in Supplementary Figure S2 as a function of the predicted day for each one of the countries which passed the initial filter.

In Supplementary Figure S3, we show the success rate of each model for each country, while different maximum relative errors are allowed (from 0.1 up to 0.5) in different predicted days (7th, 14th and 21st). In Supplementary Figure S4, we show the success rate of each model for the mean of all the predictions of all countries.

Both 2020 and 2021 are represented for each country but Sweden.

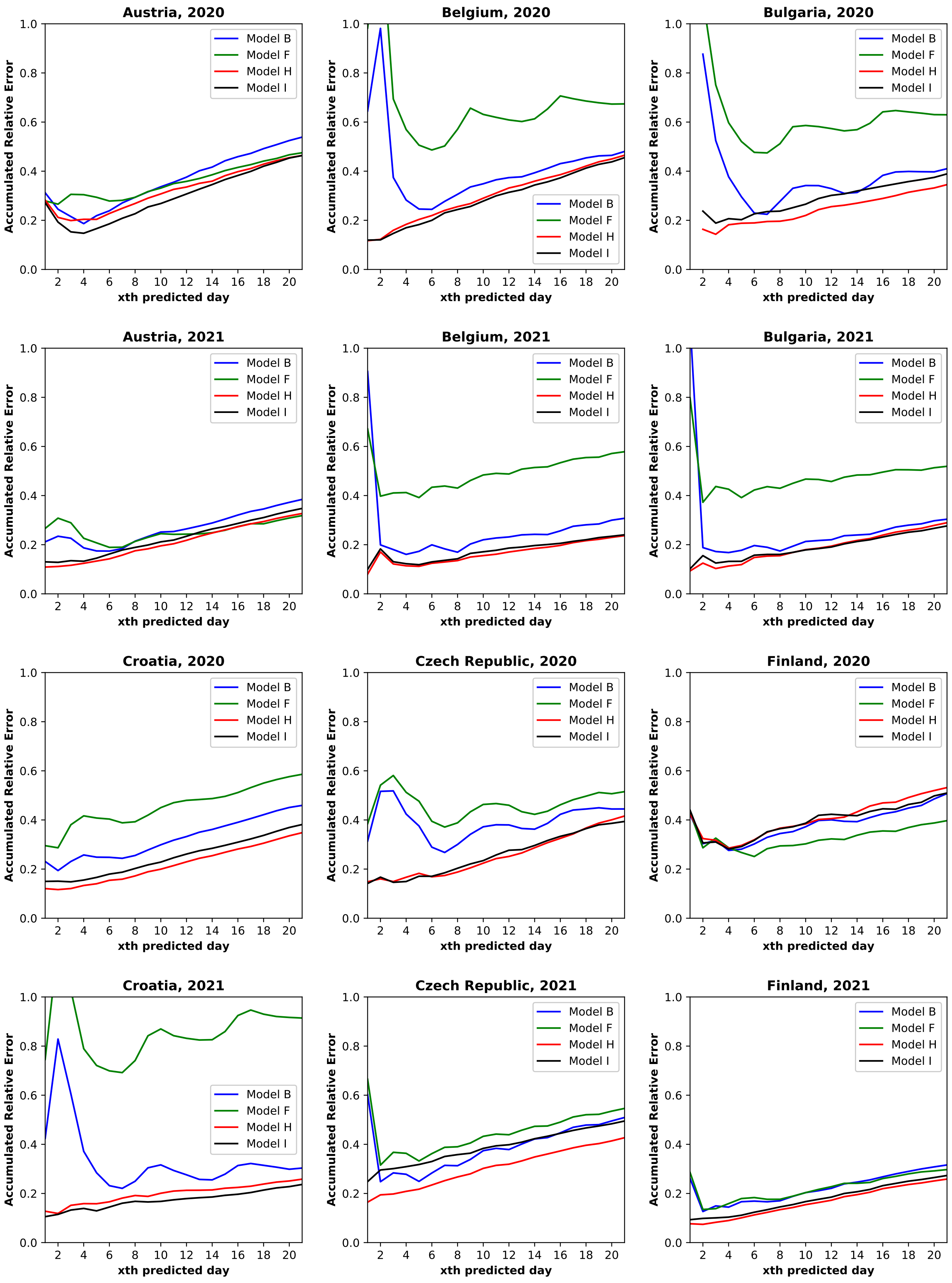
Austria, 2020
Belgium, 2020
Bulgaria, 2020
Austria, 2021
Belgium, 2021
Bulgaria, 2021
Croatia, 2020
Czech Republic, 2020
Finland, 2020
Croatia, 2021
Czech Republic, 2021
Finland, 2021
Accumulated Relative Error
xth predicted day
Model B
Model F
Model H
Model I

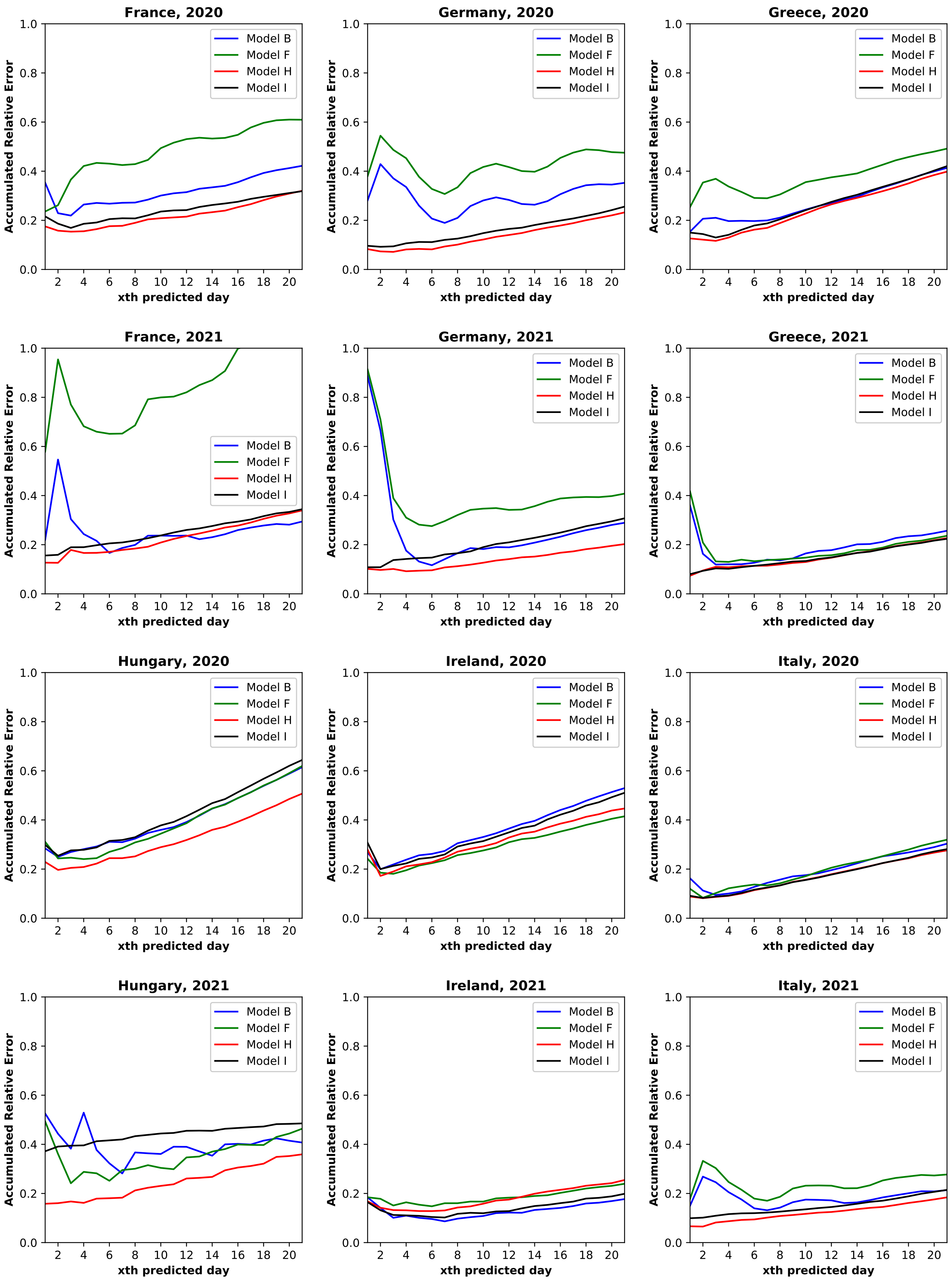
France, 2020
Germany, 2020
Greece, 2020
France, 2021
Germany, 2021
Greece, 2021
Hungary, 2020
Ireland, 2020
Italy, 2020
Hungary, 2021
Ireland, 2021
Italy, 2021
Model B
Model F
Model H
Model I
Accumulated Relative Error
xth predicted day

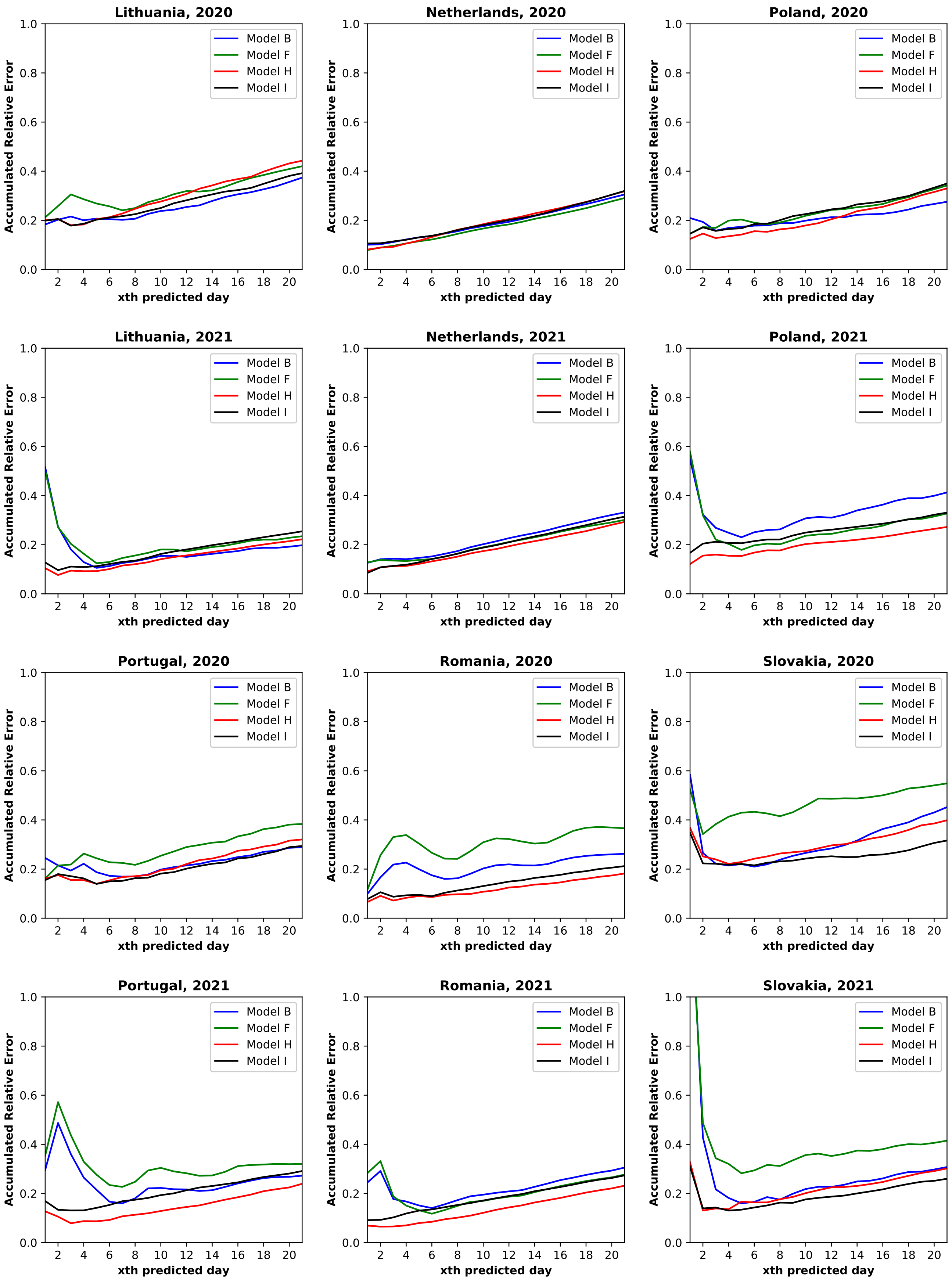
Lithuania, 2020
Netherlands, 2020
Poland, 2020
Lithuania, 2021
Netherlands, 2021
Poland, 2021
Portugal, 2020
Romania, 2020
Slovakia, 2020
Portugal, 2021
Romania, 2021
Slovakia, 2021
Accumulated Relative Error
xth predicted day
Model B
Model F
Model H
Model I

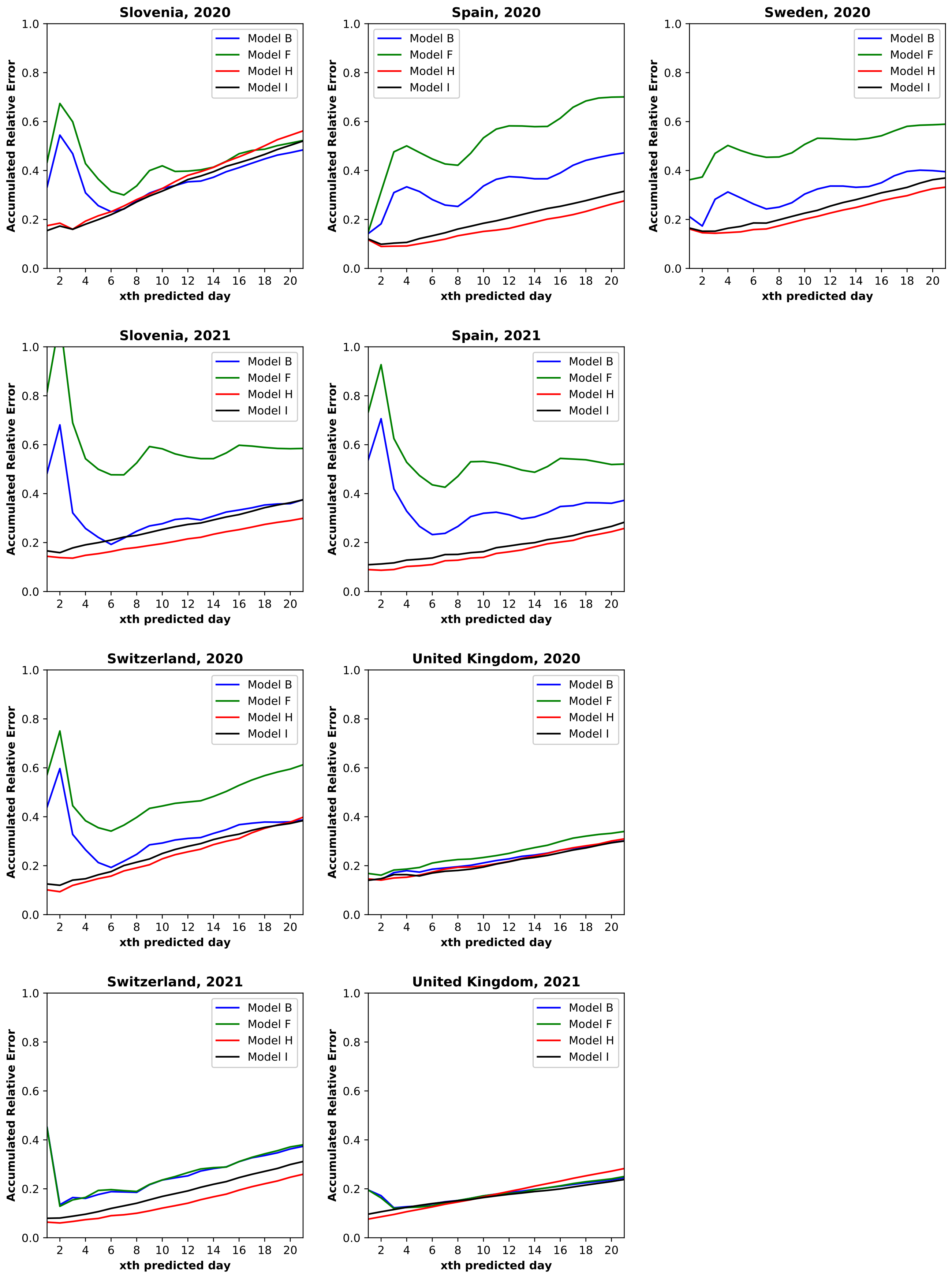

Supplementary Figure S2. Accumulated relative error as a function of the xth day of the prediction for all of the 23 countries in the study for 2020 and 2021.

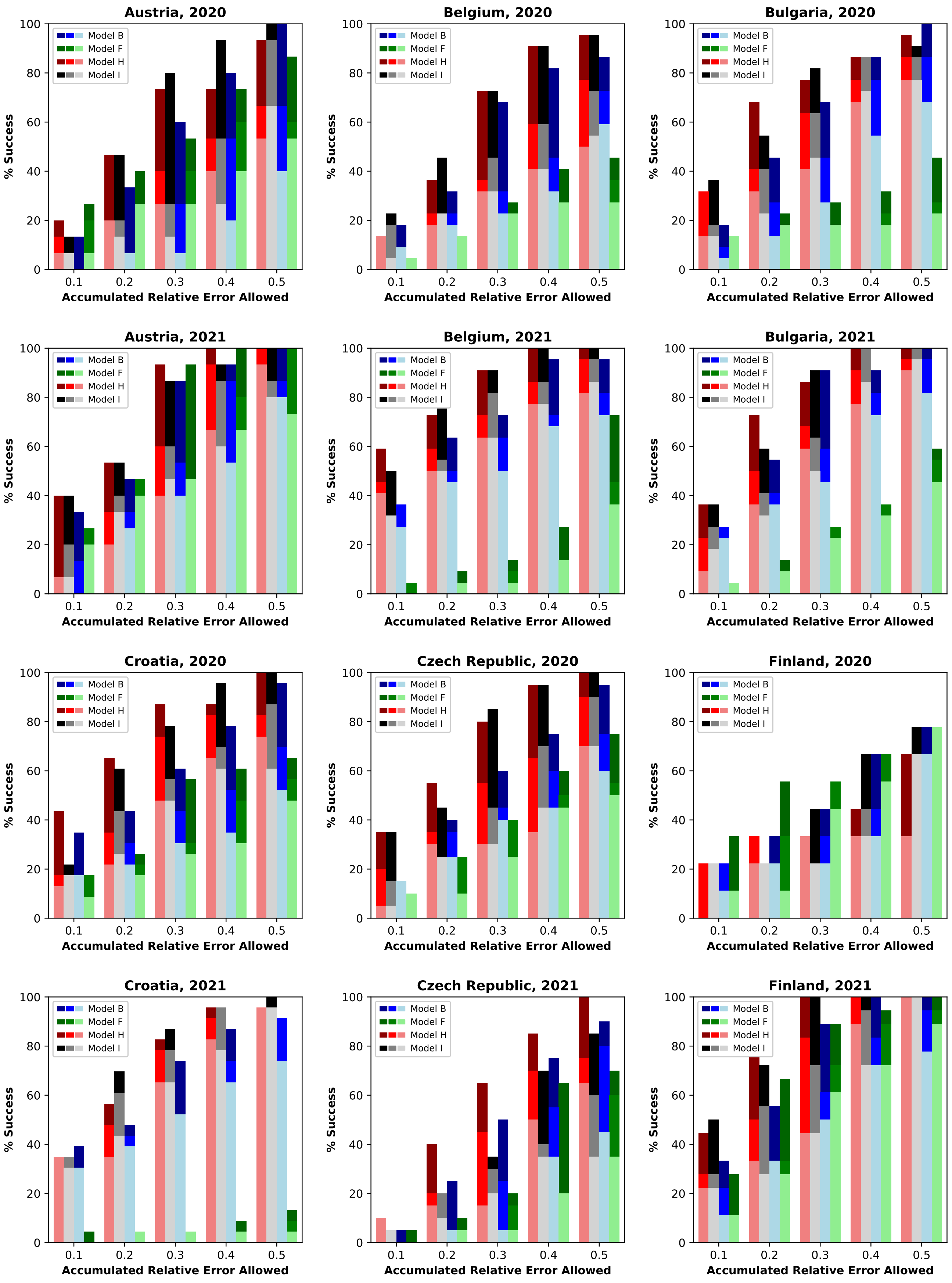
Austria, 2020
Belgium, 2020
Bulgaria, 2020
Austria, 2021
Belgium, 2021
Bulgaria, 2021
Croatia, 2020
Czech Republic, 2020
Finland, 2020
Croatia, 2021
Czech Republic, 2021
Finland, 2021
Model B
Model F
Model H
Model I
% Success
Accumulated Relative Error Allowed
0
20
40
60
80
100
0.1
0.2
0.3
0.4
0.5

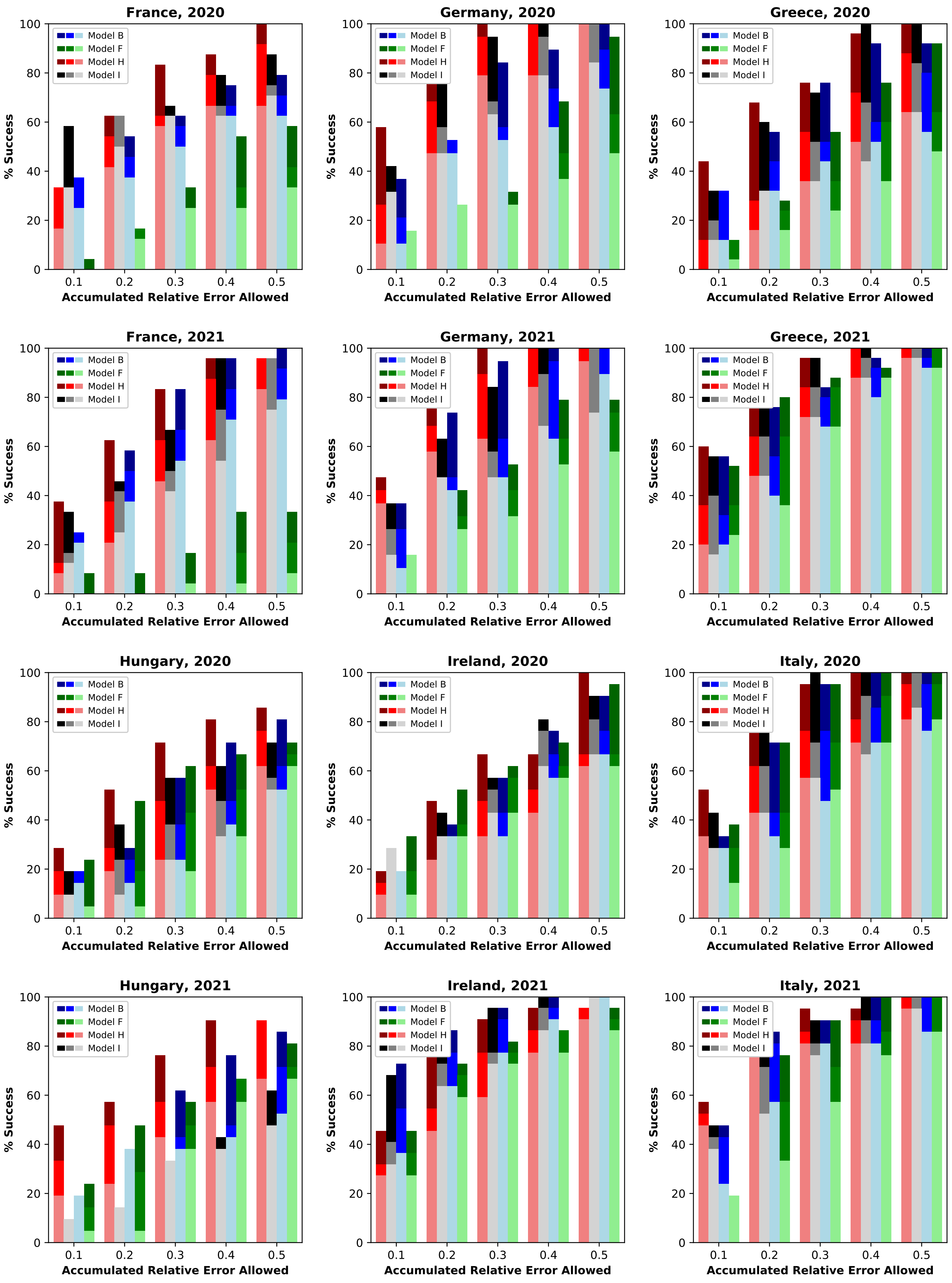
France, 2020
Germany, 2020
Greece, 2020
France, 2021
Germany, 2021
Greece, 2021
Hungary, 2020
Ireland, 2020
Italy, 2020
Hungary, 2021
Ireland, 2021
Italy, 2021
Model B
Model F
Model H
Model I
% Success
Accumulated Relative Error Allowed
0
20
40
60
80
100
0.1
0.2
0.3
0.4
0.5

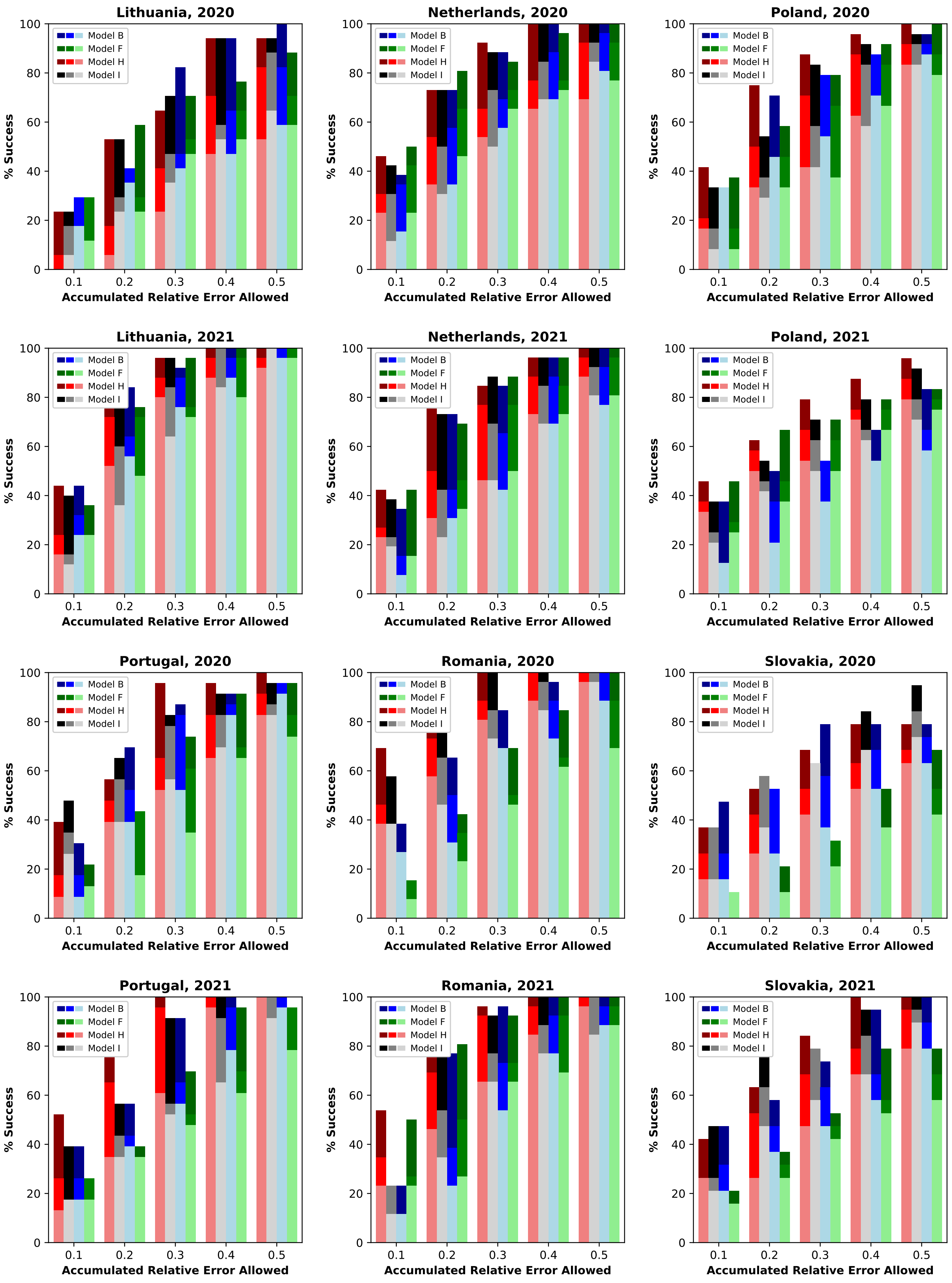
Lithuania, 2020
Netherlands, 2020
Poland, 2020
Lithuania, 2021
Netherlands, 2021
Poland, 2021
Portugal, 2020
Romania, 2020
Slovakia, 2020
Portugal, 2021
Romania, 2021
Slovakia, 2021
Model B
Model F
Model H
Model I
% Success
Accumulated Relative Error Allowed
0
20
40
60
80
100
0.1
0.2
0.3
0.4
0.5

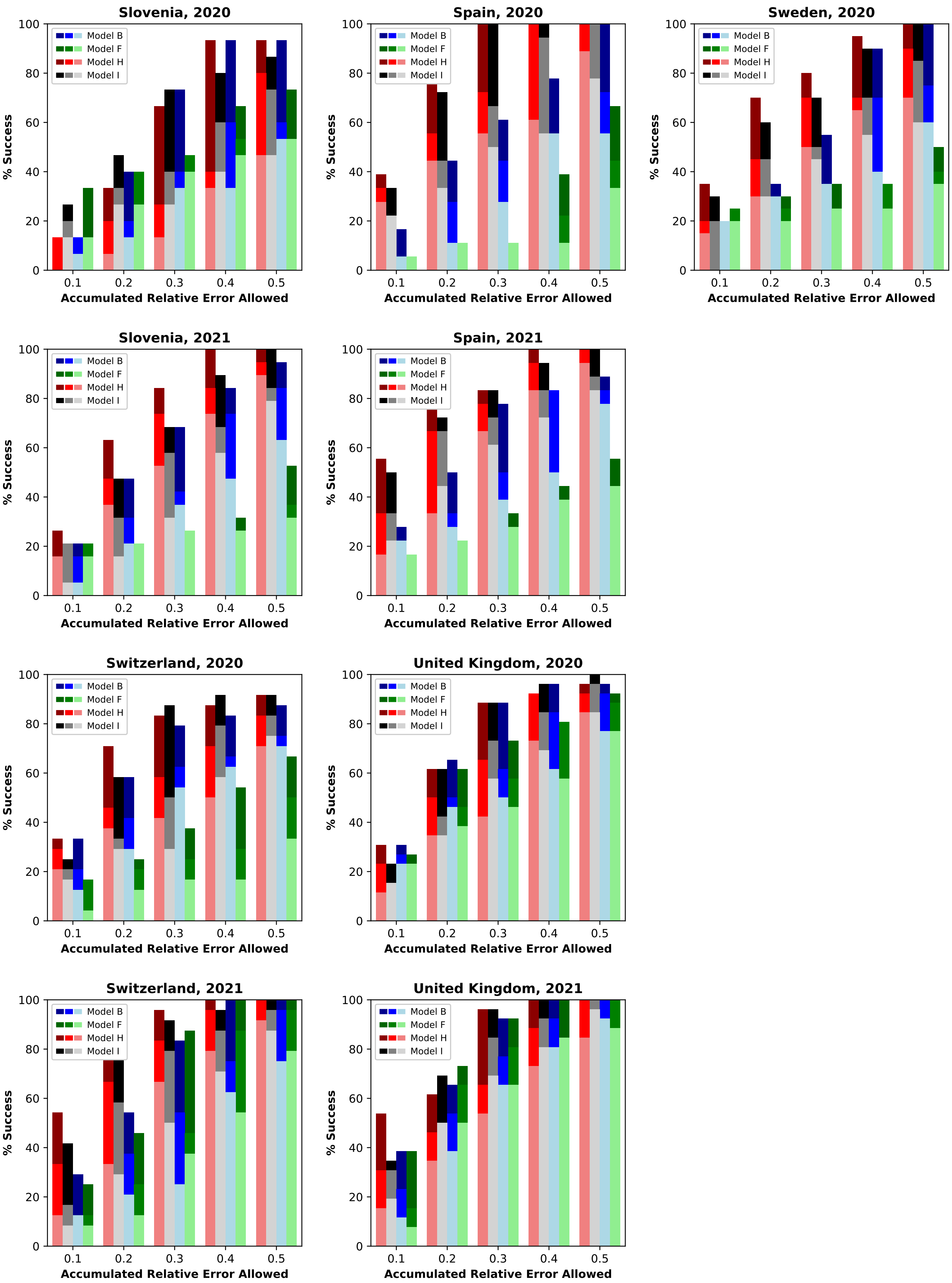

Supplementary Figure S3. Success rate for each of the model when allowing a certain error for the averaged accumulated relative error for all countries in the study, for 2020 and 2021. Lighter colours correspond to closer predicted days (7th, 14th and 21st).

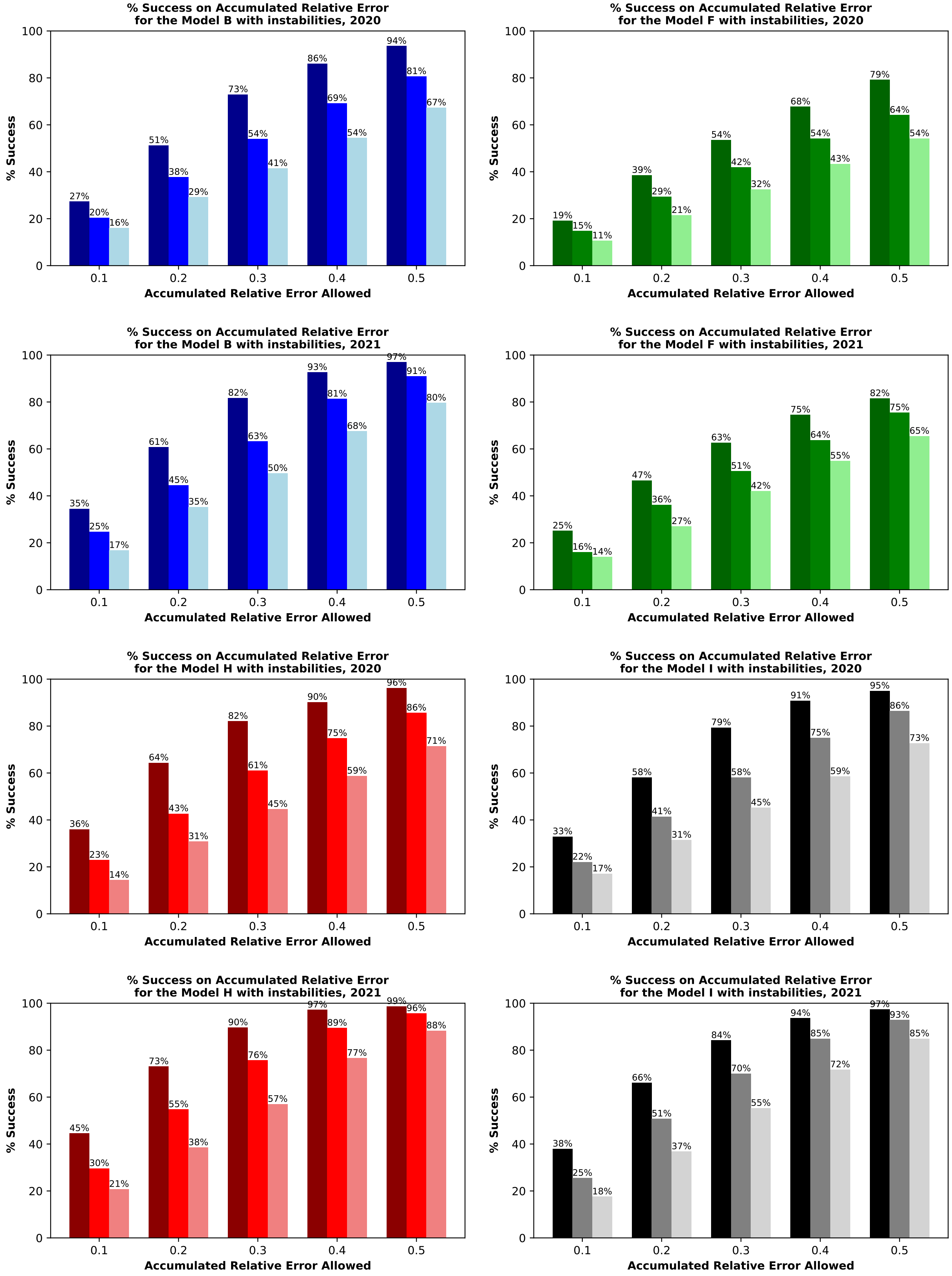

Supplementary Figure S4. Success rates for the 7th, 14th and 21st predicted days as a function of an allowed accumulated relative error for all the 4 prediction methods and 2020 and 2021.

4 Prediction filter

For every Tuesday and Saturday (2020) and every country, a filter is applied to determine whether the prediction can be reliable or not. If the average number of cases in the last 7 days is lower than 100 per day, then no prediction is made (marked in gray in Supplementary Table S2). If that is not the case, the prediction for the next 21 days is made. However, some of these predictions can be considered unstable (in red in Supplementary Table S2) if they highly depend on the number of back-days (N in the main manuscript) that is used for the fitting. The same is done with the same days of 2021, in this case Wednesday and Sunday, in Supplementary Table S3.

	AT	BE	BG	HR	CZ	FI	FR	DE	EL	HU	IE	IT	LT	NL	PL	PT	RO	SK	SI	ES	SE	CH	UK
01/09/2020																							
05/09/2020																							
08/09/2020																							
12/09/2020																							
15/09/2020																							
19/09/2020																							
22/09/2020																							
26/09/2020																							
29/09/2020																							
03/10/2020																							
06/10/2020																							
10/10/2020																							
13/10/2020																							
17/10/2020																							
20/10/2020																							
24/10/2020																							
27/10/2020																							
31/10/2020																							
03/11/2020																							
07/11/2020																							
10/11/2020																							
14/11/2020																							
17/11/2020																							
21/11/2020																							
24/11/2020																							
28/11/2020																							

Supplementary Table S2. Table of days without predictions (grey) or unstable predictions (red) for each country with their country codes for 2020.

	AT	BE	BG	HR	CZ	FI	FR	DE	EL	HU	IE	IT	LT	NL	PL	PT	RO	SK	SI	ES	SE	CH	UK
01/09/2021																							
05/09/2021																							
08/09/2021																							
12/09/2021																							
15/09/2021																							
19/09/2021																							
22/09/2021																							
26/09/2021																							
29/09/2021																							
03/10/2021																							
06/10/2021																							
10/10/2021																							
13/10/2021																							
17/10/2021																							
20/10/2021																							
24/10/2021																							
27/10/2021																							
31/10/2021																							
03/11/2021																							
07/11/2021																							
10/11/2021																							
14/11/2021																							
17/11/2021																							
21/11/2021																							
24/11/2021																							
28/11/2021																							

Supplementary Table S3. Table of days without predictions (grey) or unstable predictions (red) for each country with their country codes for 2021.
